# The effect of particle agglomeration on the formation of a surface-connected compartment induced by hydroxyapatite nanoparticles in human monocyte-derived macrophages^☆^

**DOI:** 10.1016/j.biomaterials.2013.10.041

**Published:** 2014-01

**Authors:** Karin H. Müller, Michael Motskin, Alistair J. Philpott, Alexander F. Routh, Catherine M. Shanahan, Melinda J. Duer, Jeremy N. Skepper

**Affiliations:** a Cambridge Advanced Imaging Centre, Dept. of Physiology, Development and Neuroscience, Anatomy Building, Cambridge University, Downing Street, Cambridge CB2 3DY, UK; b Dept. of Chemical Engineering and Biotechnology, New Museum Site, Pembroke Street, Cambridge CB2 3RA, UK; c Cardiovascular Division, Kings College London, The James Black Centre, 125 Coldharbour Lane, London SE5 9NU, UK; d Dept. of Chemistry, Cambridge University, Lensfield Road, Cambridge CB2 1EW, UK

**Keywords:** Hydroxyapatite nanoparticles, Agglomeration, Aggregation, Cytotoxicity, Macrophages, Surface-connected compartment

## Abstract

Agglomeration dramatically affects many aspects of nanoparticle–cell interactions. Here we show that hydroxyapatite nanoparticles formed large agglomerates in biological medium resulting in extensive particle uptake and dose-dependent cytotoxicity in human macrophages. Particle citration and/or the addition of the dispersant Darvan 7 dramatically reduced mean agglomerate sizes, the amount of particle uptake and concomitantly cytotoxicity. More surprisingly, agglomeration governed the mode of particle uptake. Agglomerates were sequestered within an extensive, interconnected membrane labyrinth open to the extracellular space. In spite of not being truly intracellular, imaging studies suggest particle degradation occurred within this surface-connected compartment (SCC). Agglomerate dispersion prevented the SCC from forming, but did not completely inhibit nanoparticle uptake by other mechanisms. The results of this study could be relevant to understanding particle–cell interactions during developmental mineral deposition, in ectopic calcification in disease, and during application of hydroxyapatite nanoparticle vectors in biomedicine.

## 1 Introduction

The mineral hydroxyapatite (HA) plays an important role during development in the formation of the bony skeleton and during adult life in bone remodelling [1]. Dysregulation of HA metabolism can lead to pathological calcification with serious clinical consequences, as seen in arterial calcification, chronic kidney disease and osteoporosis [2,3]. Calcification has been regarded as a late-stage, irreversible ‘by-product’ of disease processes, especially in arterial disease. However, more recently the process of cell-mediated ectopic HA deposition is thought to be involved in the early stages of many mineral deposition diseases [4–7], opening up the possibility that the inhibition or even reversal of calcification by therapeutic intervention may be feasible and of clinical benefit. Apart from its importance in normal and pathological biological processes, HA is also finding increasing use in numerous bioengineering applications. Nanoparticles (NPs) of HA, other calcium phosphate species and HA composites are being explored as vehicles for drug targeting, for transfection, as bone scaffolds and implant coating materials [8–10]. A thorough knowledge of all aspects of HA-cell interactions – such as their mode of uptake, cytotoxicity, intracellular trafficking, biostability, inflammatory potential etc. – are therefore of prime importance for understanding natural/pathological processes as well as for safe application of HA vectors in biomedicine.

In a previous study, we examined the interaction of human monocyte-derived macrophages (HMMs) with a range of HA nano- and micro-particles designed as potential slow-release vectors for vaccine applications. We found that their cytotoxicity varied considerably and correlated strongly with the degree of particle uptake. However, the degree of uptake did not correlate directly with any of the primary particle characteristics, such as size, aspect ratio, surface area, or zeta potential [11]. Unexpectedly, at higher concentrations the HA NPs were found sequestered within a large convoluted membrane-bound labyrinth that could occupy more than 50% of the cell's volume. Three dimensional image reconstruction showed that this branched membrane compartment was inter-connected and not truly intracellular as it was open to the extracellular space at multiple points of the cell surface [12]. The formation of a similar compartment was first described by Kruth et al. in macrophages exposed to acetylated low-density lipoprotein (LDL) or microcrystalline cholesterol and was named the surface-connected compartment, or SCC [13]. Subsequently, the SCC was also observed following exposure of macrophages to aggregated LDL, latex, gold or polystyrene nanoparticles [14–16]. Initially thought to be a macrophage-specific compartment for sequestering hydrophobic particles [13–16], we showed that formation of the SCC can be triggered by hydrophilic HA NPs and may occur in a range of cell types [12]. The function of the SCC is still unknown. We propose its formation may be a protective mechanism, an attempt to ‘shield’ themselves and surrounding cells and tissues from damage by rapid sequestration of particle agglomerates or aggregates.

At present, a definitive characterization of the particle properties that induce SCC formation is still lacking. The wide range of NPs able to induce it, speaks against a particular chemical determinant being the trigger. Observations from previous studies and by us seemed to indicate that the formation of the SCC is dependent on NP concentration [13–16]. Most of the early studies exposed cells to high concentrations of potentially aggregated materials, such as acetylated LDL, microcrystalline cholesterol, hydrophobic latex beads or polystyrene particles, although the degree of their aggregation was not measured in any of these studies. This led to our present hypothesis that the common denominator that governs SCC formation is particle agglomeration.

The formation of large NP complexes in suspension is frequently referred to as ‘aggregation’ or ‘agglomeration’. In this work, we will refer to the HA NP complexes as ‘agglomerates’, indicating that the NPs are held together by fairly weak forces and can be easily dispersed by ultrasonication or the addition of dispersants. In contrast, we would understand NP ‘aggregates’ to be held together by stronger, covalent bonds. Particle agglomeration is influenced by a number of factors, including primary particle characteristics as well as the properties of the medium that the particles are suspended in. According to the classical DLVO (Derjaguin–Landau–Verwey–Overbeek) model, the stability of NPs in suspension depends on the sum of the attractive and repulsive forces between the particles [17–19]. In biological culture media, the classical DLVO model is often unsuitable for predicting particle agglomeration due to their fairly high salt concentrations [20]. In addition, the avid binding of serum proteins to NPs can mask particle surface charges and alter their agglomeration and biological behaviour [21,22]. This underlines the importance of measuring NP agglomeration behaviour in biologically relevant media [23].

In this study, we wanted to test the hypothesis that NP agglomeration governs the formation of the SCC in macrophages. We synthesized two species of HA NPs (non-autoclaved and autoclaved) which results in chemically identical NPs with slightly different shapes and primary particle sizes. Subsequently, two strategies were used to control HA NP agglomeration post-synthesis: 1) coating the HA NPs with citrate to increase inter-particle repulsion; or 2) adding the non-cytotoxic dispersing agent Darvan 7N (Tsodium polymethacrylate). Dynamic light scattering (DLS) and a SEM/filtration method were used to assess the degree of HA NP agglomeration. Subsequently, NP agglomeration was correlated with particle uptake, cytotoxicity and SCC formation in HMMs.

## 2 Materials and methods

### 2.1 Synthesis of HA NPs

HA NPs were prepared by adding 2 L of 150 mm calcium chloride (CaCl_2_·2H_2_O) to 2 L of 100 mm tri-sodium orthophosphate dodecahydrate (Na_3_PO_4_·12H_2_O) and stirring for 1 h at RT. The resulting precipitate was allowed to settle for 24 h before thorough washing with deionised water (DIW). This HA is referred to as non-autoclaved, non-citrated (NANC). Then, HA NPs were autoclaved at 121 °C for 20 min to obtain the autoclaved HA NPs (ANC = autoclaved, non-citrated). The concentrations of these suspensions were established by determining the weight of an aliquot after drying at 60 °C for 24 h.

Citrated HA NPs were prepared by bringing the non-citrated HA preparations to a concentration of about 30 mg/ml and adding sufficient tri-sodium citrate to obtain a concentration of 200 mm. The citrate-containing suspensions were stirred at RT for 30 min, transferred to dialysis tubing (MW cut-off 10 kDa) and dialysed against a large excess of DIW for at least 24 h. The concentrations of these suspensions were determined as described above. These preparations are referred to as non-autoclaved, citrated (NAC) and autoclaved citrated (AC). All HA NP preparations were stored as aqueous suspensions at 4 °C.

Dry powders were used for XRD and BET analysis and for ICP-OES. The fairly low temperature of 60 °C (24 h) was used for drying in order not to change the NP characteristics (crystallinity, specific surface area) during the drying process.

### 2.2 Dispersion of HA NPs with Darvan 7

Darvan 7N (D7, sodium polymethacrylate, R.T. Vanderbilt Inc., USA) was supplied as a solution in water with 25% ± 2% total active solids. HA NPs were dispersed by addition of 0.125% D7 to NP suspensions as indicated. The NPs suspensions were incubated with D7 for at least 24 h at RT before addition to the cells or DLS agglomeration measurements.

### 2.3 Measurement of primary particle characteristics

Particle size analysis of all HA NP preparations was performed by BF-TEM. Particle suspensions were applied to 400 mesh Cu film grids and carbon-coated. Grids were viewed using a FEI Tecnai G2 electron microscope operating at 120 keV. Paired short- and long-axis measurements were taken of *n* = 100 particles from each preparation to give length, width and aspect ratio estimates. Selected-area diffraction patterns (SAED) were obtained at 120 keV at spot size 7.

A Philips PW1820 diffractometer with a copper target was used to obtain X-ray powder diffraction data. Spectra were obtained for 2*θ* positions between 10° and 80° with a step size of 0.05° and a dwell time of 35 s per step. Phase identification was performed by comparison with reference spectra using X-Pert High Score software.

The specific surface area of the HA powders was determined by the Brunauer–Emmett–Teller (BET) method using a surface area analyser (TriStar 3000, Micromeritics) after drying at 60 °C and degassing by vacuum evaporation. Pore surface areas and pore diameters were calculated using the BJH method (Barrett, Joyner and Halenda).

### 2.4 Zeta potential measurements

The surface charge of HA NPs was measured in suspensions of 250 μg/ml in 5 mm KNO_3_ at 20 °C and pH 7.0 using a phase amplitude light-scattering (PALS) Zeta potential analyser (Brookhaven ZetaPALS). The Smoluchowski model was fitted to 5 cycles of electrophoretic mobility (EPM) measurements and 10 replicates were taken for each sample to estimate the mean and the standard error of mean (SEM). Strictly speaking, the Smoluchowski model assumes the measured particles to be rigid spheres and therefore, the calculated zeta potentials of the HA NPs are approximate values only. However, for the sake of comparison with other studies, we chose to state zeta potential values (given in mV) rather than mobility measurements.

### 2.5 Dynamic light scattering (DLS)

The size distribution profile of HA NP suspensions was measured by DLS using an integrated Brookhaven system. HA NP suspensions were prepared at 250 μg/ml in DIW or the cell culture medium Macrophage Serum-Free Medium (Mø-SFM, Invitrogen). As indicated, D7 was added at a concentration of 0.125% for 24 h prior to measurements. A sample volume of 3 ml was measured in a quartz cuvette at 20 °C and 10 measurements of 30 s duration each were recorded for each sample.

### 2.6 SEM/filtration method

HA NP suspensions were prepared in Mø-SFM at 125 μg/ml in the absence or presence of 0.125% D7, briefly sonicated and incubated for 24 h. After brief vortexing, the particle suspensions were left to agglomerate for ∼15 min. Three millilitres of particle suspension were passed through 3 μm membrane filters (13 mm ø Nuclepore Track Etch Membranes, Whatman) using a syringe and filter holders (13 mm ø Pop Top membrane holders, Whatman). Membranes were rinsed with 2 ml of DIW to remove medium components and subsequently dried at 60 °C for 30 min. Then, filters were mounted on aluminium stubs for SEM using sticky carbon pads (Agar Scientific) and gold-coated using an Emitech K575X coater. Samples were viewed in a Philips XL-30 FEG-SEM operated at 5 kV using the secondary electron detector.

### 2.7 Isolation and culture of HMMs

Mature human macrophages (HMMs) were obtained by *in vitro* culture of human monocytes isolated from human buffy coat residues (National Blood Service, Colindale, UK) as described previously [24]. Briefly, monocytes were enriched by centrifugations on Ficoll and Percoll density gradients and seeded on tissue culture plates using Mø-SFM supplemented with 100 U/ml penicillin and 100 μg/ml streptomycin. After incubation for 1 h at 37 °C, cells were washed twice with PBS to remove any remaining non-adherent cells and monocytes were cultured at 37 °C in humidified air/5% CO_2_ for at least 6–7 days prior to experiments renewing the culture medium twice a week. For the cell exposure experiments, HA NPs were diluted from their aqueous stock solutions into cell culture medium (Mø-SFM) to the concentrations stated in the text.

### 2.8 HA-uptake by HMMs

Mature HMMs were incubated with 125 μg/ml HA NPs in Mø-SFM for 1 h at 37 °C or 4 °C in the absence or presence of 0.125% D7. Following the incubations, cells were washed three times with 0.9% saline and lysed in 0.5% Triton X100/DIW. Lysates were stored at −20 °C. For the analysis of cell-associated calcium, samples were diluted in 5% HNO_3_ and calcium concentration was measured by inductively-coupled plasma optical emission spectrometry (ICP-OES, Jobin Yvon JY2000-2) at 393.366 nm. Calcium standards were used for external calibrations in the range of 0.1–10 ppm. All dilutions were performed gravimetrically to avoid pipetting errors. Cellular protein concentration was analysed in separate wells using a standard Lowry assay and results were expressed as ppm Ca/mg protein.

### 2.9 Cytotoxicity

Viability of HMMs was assessed in 48-well tissue culture plates using the MTT (3-(4, 5-dimethylthiazol-2-yl)-2,5-diphenyl tetrazolium bromide) assay. Following incubations, sample solutions were removed and cells were incubated for 2 h at 37 °C with 0.5 mg/ml MTT in cell culture medium. After removing the dye solution, 500 μl of DMSO was added to each well. Plates were incubated for 15 min at RT to dissolve the dye, centrifuged at 500 g to sediment any floating cells or particles, and 200 μl of supernatant dye extract was transferred to 96 well plates. Absorbance was measured at 570 nm using an ASYS HiTech Expert Plus plate reader and cell viability was expressed as a percentage based on a no additions (NA) control. In the experiments, where HA NP agglomerates were dispersed with Darvan 7, an additional control was used, which consisted of Mø-SFM containing 0.125% D7.

### 2.10 Bright-field transmission electron microscopy (BF-TEM)

Following incubation with HA NPs as indicated, cells were washed three times in 0.9% saline to remove excess particles. Then, samples were fixed in 3% glutaraldehyde/1% formaldehyde in 0.05 m sodium cacodylate buffer pH 7.2 for 2 h at 4 °C. Fixed cells were scraped and washed 3 times in 0.05 m sodium cacodylate buffer. Samples were post-fixed for 1 h at RT in 1% osmium tetroxide containing 1.5% potassium ferricyanide in 0.05 m sodium cacodylate buffer pH 7.4. After rinsing 3 times in DIW to remove any buffer salts, cells were dehydrated by multiple exchanges in progressively higher ethanol concentrations up to 100%, followed by two exchanges in dry 100% acetonitrile. Samples were then incubated in 50% acetonitrile 50% Quetol epoxy resin overnight. This was followed by four daily changes of resin. The resin mixture contained: 8.75 g Quetol 651 (Agar), 13.75 g Nonenyl Succinic Anhydride (NSA) hardener, 2.5 g Methyl-5-Norbornene-2,3-Dicarboxylic Anhydride (MNA) hardener and 0.62 g Benzyldimethylamine (BDMA) catalyst. They were then cured at 60 °C for 48 h. Thin sections (60–90 nm) were cut using a Leica Ultracut UCT ultramicrotome and collected on bare 300 mesh copper grids and were not post-stained. Grids were viewed using a FEI Tecnai G2 in bright field mode operated at 120 keV and using a 10 mm objective aperture to improve contrast.

### 2.11 Statistics

Quantitative toxicity data were analysed using Analyse-it™ software embedded in Excel. Variations between groups were established using a 1-way ANOVA and a LSD *post hoc* test was used to identify homogeneous groups. *p* values ≤0.01 were considered statistically significant.

## 3 Results

### 3.1 HA particle morphology

**Fig. 1:**
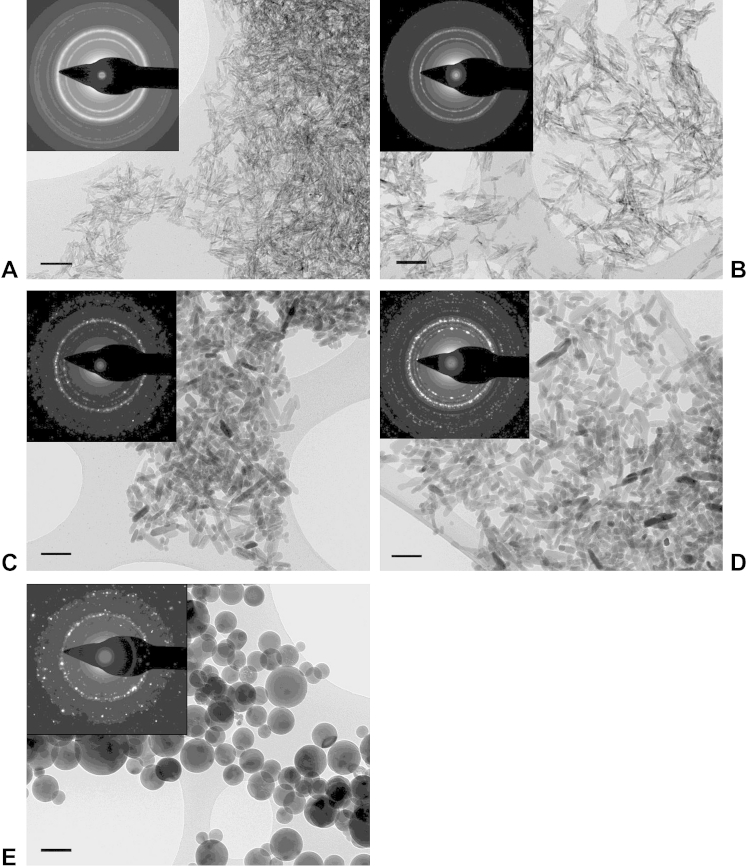
BF-TEM of HA NPs. A) NANC; B) NAC; C) ANC; D) AC; E) Sigma–Aldrich HA nano-powder. Scale bars are 100 nm. Inserts are the respective SAED patterns.

Synthesis of HA NPs by precipitation resulted in the formation of fine needle- to plate-like nanoparticles (Fig. 1A; NANC = non-autoclaved, non-citrated). Autoclaving at 120 °C for 20 min resulted in a visible shape change. The NPs were longer, thicker and more lozenge-shaped (Fig. 1C; ANC = autoclaved, non-citrated). This is consistent with results reported in earlier studies [11,25]. Citration of the non-autoclaved (NAC = non-autoclaved, citrated) or autoclaved (AC = autoclaved, citrated) HA NPs did not visibly affect their morphology (Fig. 1B and D, respectively). A synthetic HA nano-powder from Sigma–Aldrich (cat no. 677418) was used as a comparison for SAED and was composed of comparatively large, round particles (Fig. 1E). The SAED patterns of the four HA NP preparations and of the Sigma–Aldrich HA standard showed that all the materials were polycrystalline with very similar d-spacing and intensity profiles (Fig. 1A–E, insets).

### 3.2 Primary particle characteristics

The primary particle characteristics of the HA NP preparations are summarized in Table 1. NANC particles were 30 nm in the long axis and 8 nm in the short axis and had an aspect ratio of 3.9. Autoclaving led to an increase in length and thickness to 54 nm in the long axis and 18 nm in the short axis and a smaller aspect ratio of 3.2. After citration, both non-autoclaved and autoclaved HA NPs were slightly shorter in the long axis, whereas the short axis remained unchanged. Phase identification by XRD analysis indicated that all four NP preparations were hydroxyapatite and did not contain any other calcium phosphate phases (Figure SI1 and SI2, Supplementary Information). Surface area and porosity analysis using the BET/BJH methods showed that all four HA NP powders were mesoporous materials (see Figure SI3, Supplementary Information). The specific surface areas of the non-autoclaved HA NPs was about 2–3 times that of the autoclaved HA NPs, whereas citration had comparatively little effect. Similarly, Lopez-Macipe et al. [26] detected no differences in XRD spectra and specific surface area in HA NPs post-citration. In contrast to post-synthesis citration, inclusion of citrate *during* HA precipitation can have significant effects on particle size and crystallinity [27,28]. In our study, the calcium content determined by ICP-OES was 34.87% for NANC and 34.90% for ANC, which is slightly lower than the value reported for stoichiometric HA (Ca_5_(PO_4_)_3_(OH), 39.89%). This is due to residual water as drying was performed at the low temperature of 60 °C. The calcium content after citration was slightly lower and was 31.93% and 31.85% for NAC and AC, respectively. The NANC and ANC particles were negatively charged and according to the Smoluchowski model had equivalent zeta potentials of −17.80 mV and −10.56 mV, respectively (see Materials and Methods section). Citration had little effect on the zeta potential of non-autoclaved HA, whereas that of non-autoclaved HA NPs became more negative. Treatment with D7 resulted in a significant increase in negative zeta potential for all HA NPs.

### 3.3 HA NP agglomerate sizes by DLS and SEM/filtration method

**Fig. 2:**
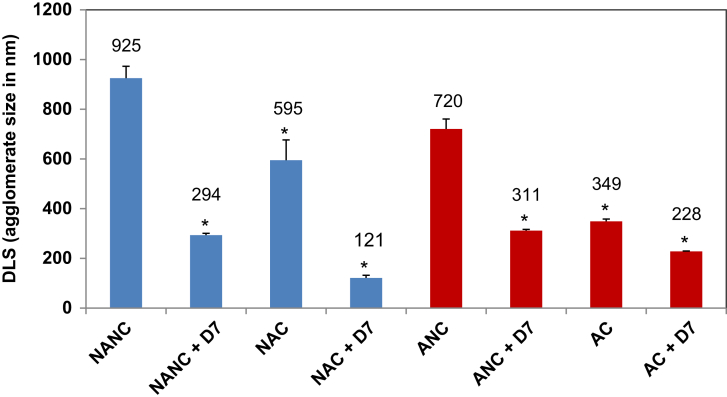
Average agglomerate sizes of HA NPs in Mø-SFM by DLS. Values shown (in nm) represent the mean ± SD of *n* = 6. *equals *p* ≤ 0.01, when comparing untreated HA NPs (NANC/ANC) to their respective counterparts after citration, addition of D7, or the combination treatment.

The agglomeration of the various HA NP preparations was measured by DLS in Mø-SFM culture medium (Fig. 2). In the absence of any method of dispersion, both NANC and ANC formed large agglomerates, with NANC agglomerates being significantly larger than those of ANC. Citration, addition of D7 or the combination of both treatments (citration + D7) all led to significant decreases in average agglomerate sizes for both non-autoclaved and autoclaved HA (Fig. 2). The addition of D7 led to a significantly larger reduction in agglomerate size than citration for non-autoclaved HA, whereas for autoclaved HA, the two methods worked equally well. For both non-autoclaved and autoclaved HA, the combination treatment (citration + D7) significantly reduced agglomerate sizes even further when compared to each method alone (Fig. 2). None of the methods achieved dispersion of HA NPs down to primary particle sizes. For comparison with other studies the agglomerate sizes of HA NPs were also measured in DIW, see Table SI1 (Supplementary Information).

**Fig. 3:**
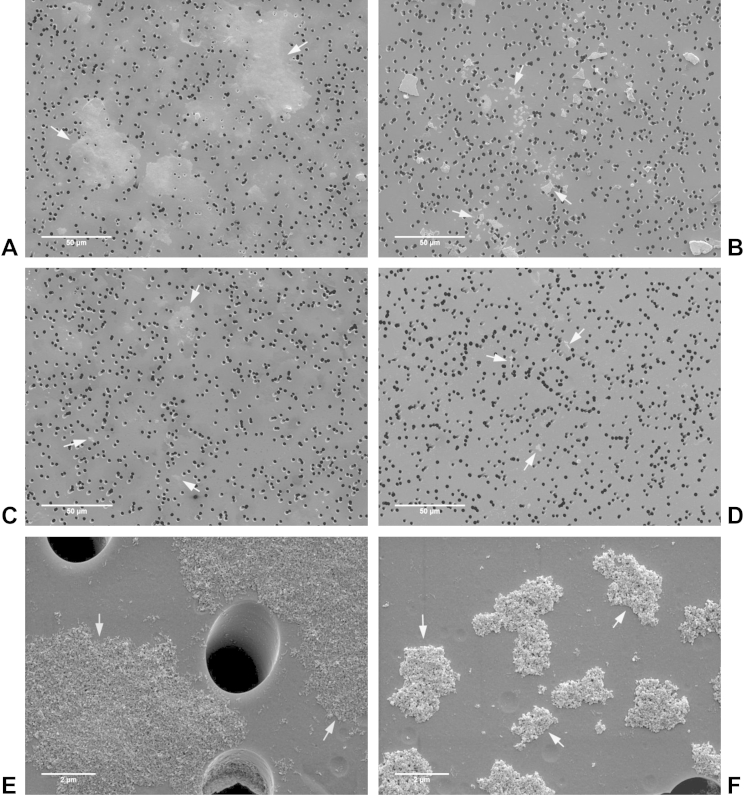
SEM of HA NP agglomerates. HA NPs were suspended in Mø-SFM at 125 μg/ml and incubated overnight. Then, suspensions were passed through membrane filters with 3 μm pores. Subsequently, filters were prepared for SEM. A) NANC; B) ANC; C) NAC; D) AC; E) NANC – detail; F) ANC – detail.

The presence of very large agglomerates can pose problems during DLS measurements. Therefore, we also employed a SEM/filtration method to examine the HA NP suspensions in cell culture medium. HA NPs were suspended in Mø-SFM at a concentration of 125 μg/ml in the absence or presence of 0.125% D7 and incubated overnight. After brief mixing, suspensions were passed through membrane filters with 3 μm pores to observe the agglomerates retained by the filters. In the absence of D7, suspensions of NANC and ANC clearly left large areas of the filters covered by agglomerates (Fig. 3A and B, arrows). In contrast, considerably fewer agglomerates were detected on the filters treated with NAC and AC suspensions (Fig. 3C and D, arrows). At larger magnification, the individual primary particles of the agglomerates were clearly visible (Fig. 3D and E, arrows). Agglomerates of NANC appeared more disperse and flat on the filters compared to agglomerates of ANC, which appeared smaller and more compact. In the presence of 0.125% D7, all four HA NP suspensions left hardly any agglomerates on the filters (Figure SI4 A–D). When isolated agglomerates were located (Figure SI4E and F), NANC and ANC agglomerates had a similar appearance compared to the agglomerates in the suspensions without D7.

If our hypothesis that NP agglomeration governs the formation of the SCC is correct, we would expect both NANC and ANC to induce an SCC in HMMs. Treatment of HA NPs by citration or addition of the dispersant D7 should reduce its formation, and the combination treatment (citration + D7) should be even more effective.

### 3.4 Cytotoxicity of HA NPs to HMMs

**Fig. 4:**
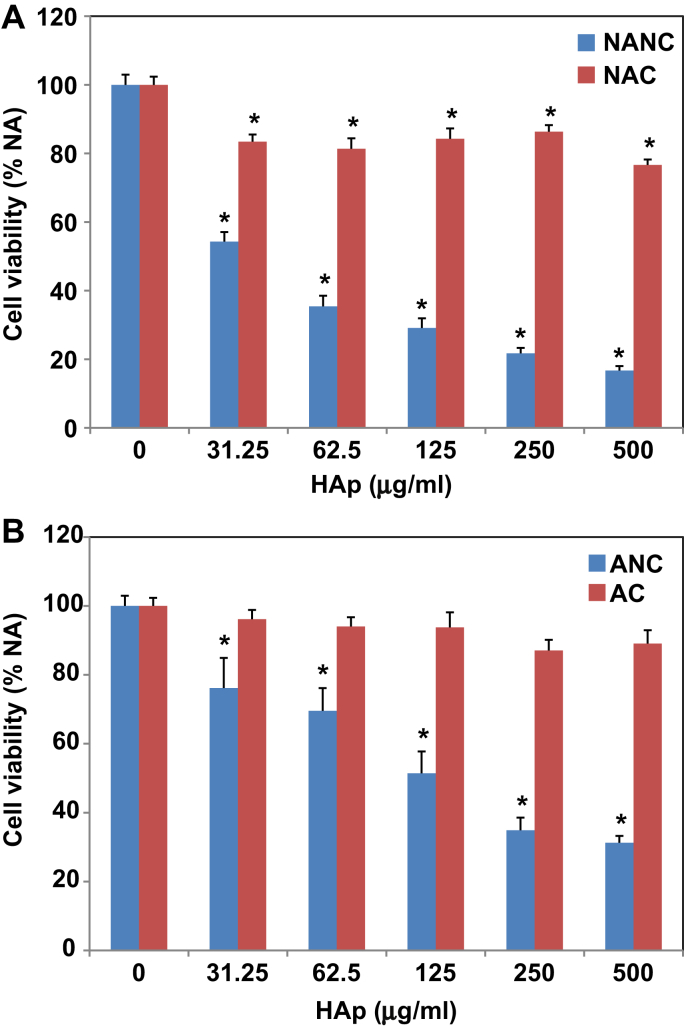
Cytotoxicity of HA NPs to HMMs. Cells were incubated with the indicated concentrations of A) non-autoclaved or B) autoclaved HA NPs for 24 h prior to measuring cell viability using the MTT assay. Values represent the mean ± SE of three experiments, each performed in triplicate (*n* = 9); *equals *p* ≤ 0.01 when compared to the NA-control.

In order to avoid overtly toxic concentrations in the cell uptake and imaging studies, we first established the cytotoxicity of the various HA NP preparations using the MTT assay in a range of 31.25–500 μg/ml (Fig. 4). After 24 h, NANC and ANC were significantly toxic to HMMs at all concentrations tested when compared to the NA-control (no additions control). NANC was significantly more toxic than ANC at all concentrations. At 24 h, the LD_50_ of NANC was ∼44 μg/ml, whereas that of ANC was ∼122 μg/ml. Citration significantly reduced the toxicity of both non-autoclaved and autoclaved HA. NAC was still significantly toxic compared to the NA-control, whereas AC became non-toxic. Consequently, HA NP top concentrations were capped at 125 μg/ml for subsequent uptake and imaging experiments.

### 3.5 Effect of HA NP concentration on SCC formation

In a previous study, SCC formation after exposure to autoclaved HA NPs was mainly observed at high NP concentrations, suggesting that SCC formation may be due to particle agglomeration [11]. We incubated HMMs with 30, 60, or 125 μg/ml HA NPs for 2 h in order to establish whether SCC formation was concentration-dependent. The shorter incubation period of 2 h was chosen to limit effects of potential uptake saturation or cytotoxicity on particle uptake.

**Fig. 5:**
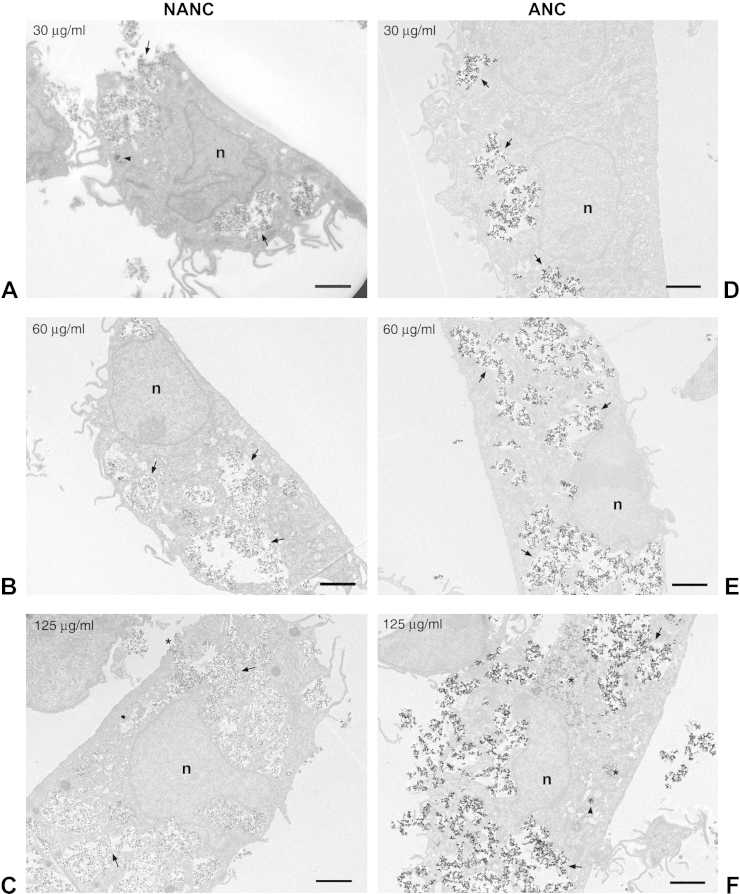
Concentration-dependence of SCC formation. HMMs were incubated for 2 h with A) 30 μg/ml; B) 60 μg/ml or C) 125 μg/ml NANC (left column) or with D) 30 μg/ml; E) 60 μg/ml or F) 125 μg/ml ANC; scale bars are 2 μm (n = nucleus).

Exposure of HMMs to increasing concentrations of NANC led to increasing particle sequestration by the HMMs (Fig. 5A–C). The particles were localized inside large, inter-connected vacuoles lined by membrane, suggestive of the SCC. With increasing concentration, the SCC appeared to occupy ever larger portions of the cells (Fig. 5A–C, arrows). In Fig. 5C an opening of the SCC to the extracellular space is visible (asterisk). Occasionally, smaller vacuoles with more densely packed NPs were present (Fig. 5A, arrowhead). A similar uptake pattern was observed for ANC (Fig. 5D–F, arrows). Again, more densely packed NPs were seen occasionally in smaller vacuoles (Fig. 5F, arrowhead). In some cells, electron-dense particles with a finer, more granular appearance were found within large and small vacuoles (Fig. 5F, asterisks), which may represent starting degradation of the NPs. Performing the same experiment with a 24 h incubation period yielded very similar results (see Figure SI5, Supplementary Information). However, after 24 h the number of damaged and dead cells in the cultures had increased at higher NANC and ANC concentrations as was to be expected from the cytotoxicity experiment (not shown). Taken together, both NANC and ANC are sequestered inside the SCC, which becomes more extensive with increasing particle concentrations.

### 3.6 Effect of agglomerate dispersion on SCC formation

Subsequently, we examined the effect of dispersing HA NP agglomerates by citration or by addition of D7 on SCC formation. HMMs were cultured for 2 h or 24 h with 125 μg/ml HA NPs, which were either untreated or had been pre-treated with 0.125% D7 for 24 h prior to addition to the cells. Again, the results for the 2 h- and 24 h-incubation periods were very similar and only those for the 24 h-incubation are shown.

**Fig. 6:**
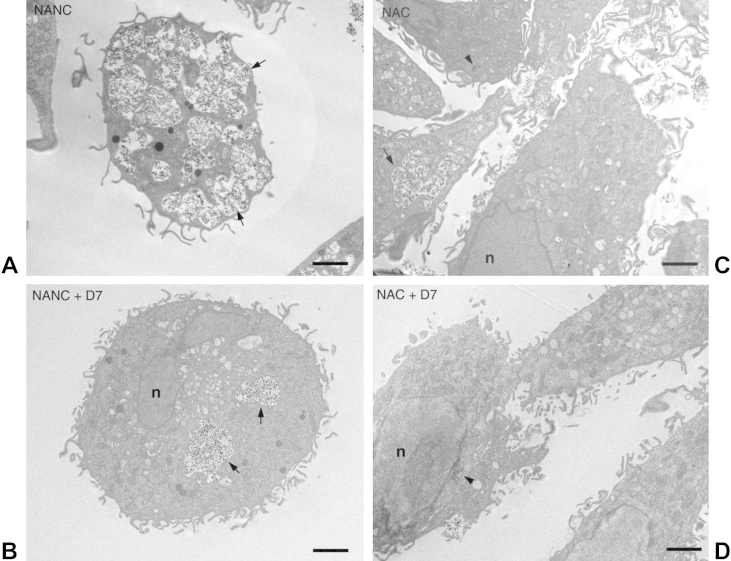
Effect of agglomerate dispersion on SCC formation by non-autoclaved HA NPs. HMMs were incubated for 24 h with 125 μg/ml A) NANC; B) NANC + D7; C) NAC; D) NAC + D7; scale bars are 2 μm (n = nucleus).

**Fig. 7:**
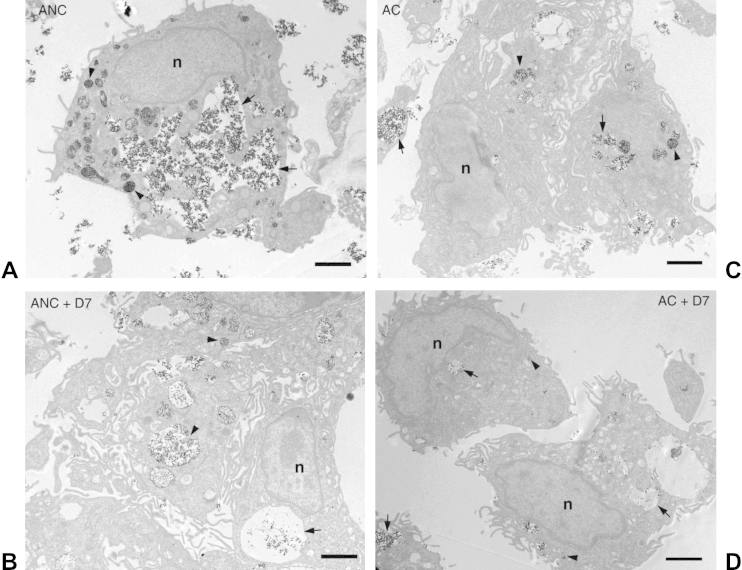
Effect of agglomerate dispersion on SCC formation by autoclaved HA NP. HMMs were incubated for 24 h with 125 μg/ml A) ANC; B) ANC + D7; C) AC; D) AC + D7; scale bars are 2 μm (n = nucleus).

Cells cultured with 0.125% D7 alone were not visibly adversely affected after 24 h incubation and showed morphology similar to NA-control cells (Figure SI6A and B). Culture with 125 μg/ml NANC for 24 h in the absence of D7 led to copious sequestration of the HA NPs into an extensive, interconnected SCC (Fig. 6A, arrows). Addition of D7 significantly reduced, but did not completely abolish particle internalization. Particles were still found in large vacuoles, which could represent large phagosomes or residual SCC (Fig. 6B, arrows). Citration had a similar effect. NAC particles could still be found in larger vacuoles suggestive of SCC (Fig. 6C, arrow), as well as in smaller vesicles with higher packing density (Fig. 6C, arrowhead). The combination of citration and addition of D7 (NAC + D7) reduced particle uptake into the SCC almost completely and particles were mostly found in small, isolated vacuoles (Fig. 6D, arrowhead). Incubation of cells with ANC resulted in excessive sequestration of the NPs into the SCC (Fig. 7A, arrows), as well as into smaller, more isolated vacuoles, where the particles were packed with higher density (Fig. 7A, arrowheads). Addition of D7 led to a considerable decrease in uptake. Particles were found in larger vacuoles, which could represent phagosomes or residual SCC (Fig. 7B, arrow). In addition, particles were sequestered in smaller vacuoles with higher packing density (Fig. 7B, arrowheads). Citration had a similar effect to D7. Uptake of AC particles was reduced, but not completely abolished. NPs were again found in larger vacuoles, suggestive of residual SCC (Fig. 7C, arrows), as well as in smaller, more isolated vacuoles with higher packing density (Fig. 7C, arrowheads). The combination of citration and D7 (AC + D7) was most effective in preventing SCC formation (Fig. 7D). Particles were almost exclusively contained within small, isolated vacuoles with loose (arrows) or dense (arrowheads) packing density.

### 3.7 Degradation of HA NPs within the SCC

**Fig. 8:**
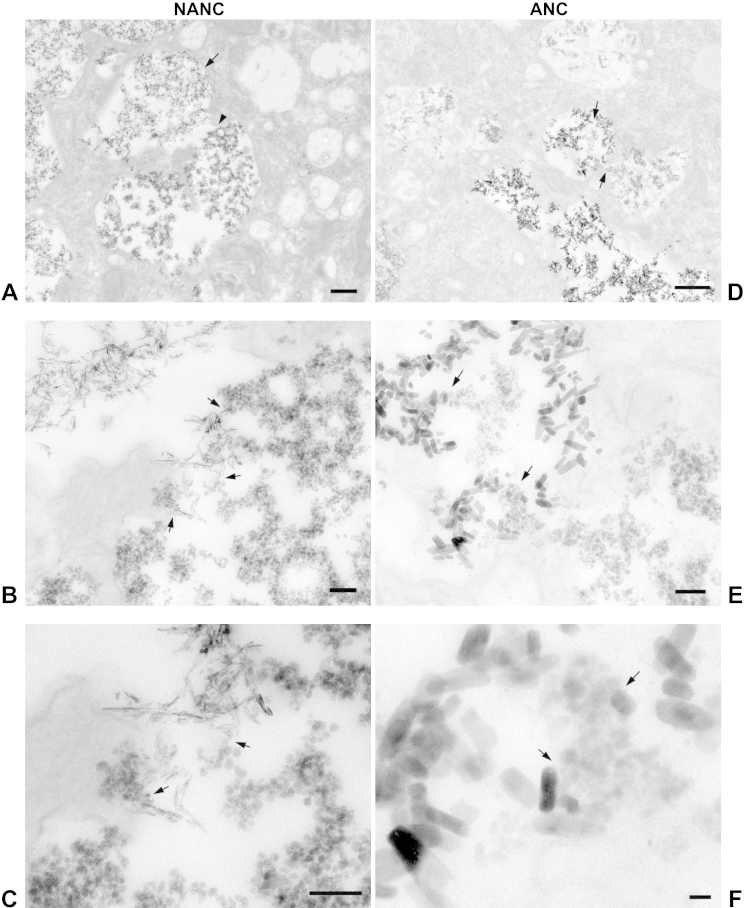
BF-TEM of HA NPs degradation within the SCC. HMMs were incubated with NANC (A–C) or with ANC (D–F) for 2 h prior to TEM processing. Scale bars are 500 nm for A and D; 100 nm for B, C and E; 20 nm for F.

During the uptake study, we observed that HA NPs sequestered by the cells within the SCC occasionally changed their appearance. After 2 h, NANC and ANC particles were found sequestered within the SCC (Fig. 8A and D, arrows). Two different particle morphologies were found in close vicinity within the same compartment and did not appear to be separated by a membrane (Fig. 8B and E, arrows). At higher magnification, the change in particle morphology from a fine needle- or plate-like shape to a smaller, more rounded appearance becomes more obvious (Fig. 8C and F). Refer to Figure SI7 (Supplementary Information) for low magnification images of these cells showing the extent of the SCC more clearly. We suggest that this change in particle morphology is due to degradation of the HA NPs within the SCC.

### 3.8 Quantification of HA NP uptake

In a previous study, particle internalization was quantified by TEM using a stereological approach [11]. In this study, we used ICP-OES to quantify cellular HA NP uptake. HMMs were incubated with 125 μg/ml HA NPs in the absence or presence of 0.125% D7 at 37 °C for 1 h prior to lysis and analysis. Cells were also incubated at 4 °C on ice to inhibit active uptake mechanisms and to monitor agglomerate binding. Again, a short incubation period was chosen to avoid effects of cytotoxicity, saturation or particle degradation.

**Fig. 9:**
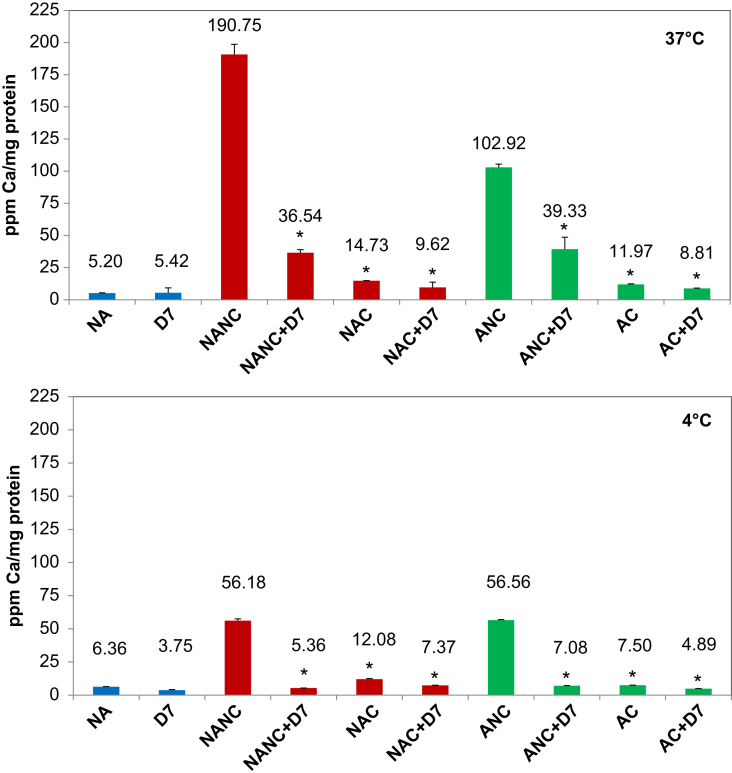
Quantification of cell-associated calcium by ICP-OES. HMMs were incubated with 125 μg/ml of HA NPs for 1 h in the absence or presence of 0.125% D7 at 4 °C or 37 °C. Then, cell-associated calcium was measured by ICP-OES. Values represent the mean ± SD of *n* = 5. *equals *p* ≤ 0.01.

Incubation with NANC and ANC at 37 °C led to a dramatic increase in cellular calcium content to levels of ∼37× and ∼20× that of control cells (NA = no additions), with NANC being significantly higher than ANC (Fig. 9). The dispersion of agglomerates by citration, addition of D7 or the combination treatment all significantly reduced particle uptake. Citration was more effective than addition of D7 for both autoclaved and non-autoclaved HA NPs. The combination treatment (citration + D7) reduced cellular Ca-content even further than D7 or citration on their own to levels of ∼2× that of untreated control cells. Incubation at 4 °C showed that the undispersed HA NPs NANC and ANC bind to a considerable degree to the cell surface as cell-associated calcium is still significantly elevated compared to untreated control cells. After dispersion by D7, citration or the combination treatment, cell-associated calcium levels are reduced to near control values indicating that only fairly small amounts of agglomerates are binding under these conditions.

### 3.9 Effect of agglomerate dispersion on cytotoxicity

**Fig. 10:**
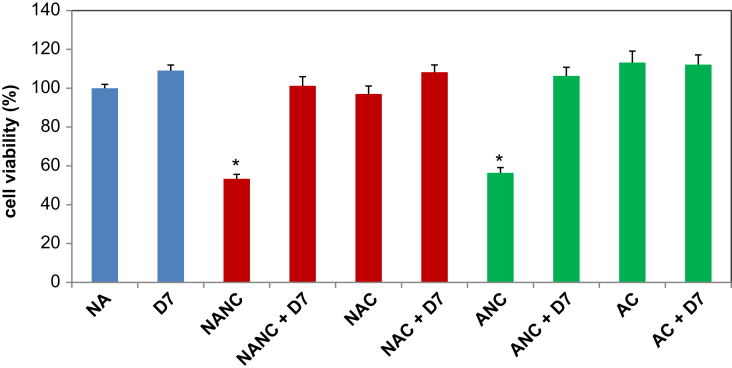
Effect of agglomerate dispersion on cytotoxicity. HMMs were incubated with 125 μg/ml HA NPs for 24 h in the absence or presence of 0.125% D7. Then, cell viability was measured using the MTT assay. Values represent the mean ± SE of 3 experiments, each performed in triplicate. *equals *p* ≤ 0.01.

We assessed cell viability after incubating the cells with 125 μg/ml HA NPs in the absence or presence of 0.125% D7 for 24 h using the MTT assay (Fig. 10). Culturing HMMs with 0.125% D7 alone for 24 h was not significantly toxic. In contrast, both NANC and ANC were significantly toxic when compared to the NA-control and reduced cell viability by about 45%. Dispersion of HA NP agglomerates by citration, addition of D7 or the combination treatment (citration + D7) inhibited this toxicity and brought cell viability back to control levels.

## 4 Discussion

In this study we showed that HA NP agglomeration governs the formation of the surface-connected compartment (SCC) in HMMs. Four species of HA NPs were synthesized by a combination of precipitation reaction, autoclaving and post-synthesis citration: NANC (non-autoclaved, non-citrated), NAC (non-autoclaved, citrated), ANC (autoclaved, not-citrated) and AC (autoclaved, citrated). In addition to post-synthesis citration, the dispersant Darvan 7 (D7) was used to control HA NP agglomeration. Particle agglomeration was then correlated with particle uptake, toxicity and SCC formation in HMMs.

### 4.1 Effect of citration and D7 on surface potential and agglomerate sizes

According to the classical DLVO model, the colloidal stability of particles in solution is determined by the sum of attractive and repulsive forces between individual particles. Attractive forces stem from van der Waals interactions, whereas repulsive forces depend on the surface charge of the particles and the thickness of the ionic double layer surrounding the particle. The thickness of the double layer depends on the ionic strength of the solution [17–19]. Dispersion of NP agglomerates in solution can be achieved by i) increasing the NP charge; ii) by adding molecular side chains to the NPs that induce steric hindrance to agglomeration; and iii) by electrosteric stabilization, which is a combination of electrostatic repulsion and steric hindrance. We employed two approaches in order to achieve HA NP dispersion: i) post-synthesis citration, or iii) addition of the dispersant D7.

The adsorption of citrate to HA is thought to proceed by ionic exchange of phosphate groups by citrate at the solid–solution interface due to the higher affinity of citrate for the Ca-sites on the HA surface [26]. This surface adsorption of citrate gives the HA NP a more negative surface potential, which in turn increases inter-particle repulsion [26,28]. In contrast, the dispersant D7 is a sodium polymethacrylate (C_4_H_5_NaO_2_)_n_ polyanion with a molecular weight of ∼13 kD. To date, D7 has not been used widely as a dispersant in biomedical applications, in spite of meeting FDA recommendations in terms of biocompatibility [FDA 21CFR 173.310]. The related compound ammonium polymethacrylate has been applied successfully to stabilize hydroxyapatite slurries for the production of ceramic bone scaffolds [29]. In aqueous solution, the D7 anions consist of extended, negatively charged polymer chains. These adsorb onto the surface of HA NPs providing steric hindrance as well as electrostatic repulsion against NP agglomeration [29,30]. Both mechanisms – the increased negative surface potential after citration as well as the electrosteric hindrance after adsorption of D7 – should be effective in dispersing HA NP agglomerates in solution. However, the relatively high salt concentrations and protein content of biological media can dramatically modify the expected agglomeration behaviour of NPs [20–22].

In our study, citration did not significantly change the negative surface potential of non-autoclaved HA NPs (−17.8 mV before, −14.48 mV after citration), whereas the negative potential of autoclaved HA NPs increased from −10.56 mV to −24.29 mV. However, ICP-OES showed that the Ca-content of non-autoclaved and autoclaved HA NPs had decreased by a similar extent after citration (2.94% and 3.05%, respectively), suggesting that similar amounts of citrate had been adsorbed by both autoclaved and non-autoclaved NPs. The fact that no increase in negative potential was measured for non-autoclaved HA after citration may be caused by agglomeration during analysis, which may have resulted in an overestimate of the potential. Alternatively, the adsorption of a similar amount of citrate onto non-autoclaved HA NPs may have resulted in a lesser increase in surface charge density as the specific surface area (BET) of non-autoclaved HA was about twice that of autoclaved HA NPs. In contrast, the addition of D7 led to significant increases in negative zeta potential for all HA NPs, rendering them more colloidally stable and reducing agglomeration. The zeta potential values cited here were measured in 5 mm KNO_3_ at pH 7.0, which may not predict the zeta potential and agglomeration tendency of the HA NPs in Mø-SFM cell culture medium. Our zeta potential measurements in Mø-SFM were inconclusive, may be due to NP agglomeration. Strong aggregation of some NP species upon suspension in cell culture medium has hampered zeta potential measurements in other studies [31,32]. Furthermore, prediction of zeta potential changes upon transfer of NPs into cell culture medium is problematical. In a study by Limbach et al., a variety of metal oxide NPs with zeta potentials in water ranging from −25 mV to +55 mV underwent dramatic zeta potential changes in cell culture medium, thought to be due to protein adsorption. Irrespective of their initial zeta potential, all NP species tested ended up with a zeta potential of about −18 mV [33]. A similar effect was observed after transfer of SPIONs (super-paramagnetic iron oxide nanoparticles) coated with various surface polymers into cell culture medium. All SPION species adsorbed a similar pattern of proteins and ended up having a zeta potential of about −12 mV. Yet, they showed significant differences in their aggregation behaviour [34]. In contrast, a range of silica NPs functionalized by different chemical groups retained a range of negative and positive zeta potentials after dispersion in cell culture medium [32]. This underlines the importance of measuring the actual agglomerate sizes of NPs in the cell culture medium used for experiments, rather than relying on zeta potential measurements to predict likely agglomeration behaviour.

In our study, untreated NANC and ANC HA NPs formed large agglomerates in Mø-SFM as measured by DLS and the SEM/filtration method. The tendency of NANC to form larger agglomerates than ANC may be due to the smaller size and the larger specific surface area of their primary particles. Citration and the addition of D7 significantly decreased the average agglomerate sizes of non-autoclaved and autoclaved HA NPs, suggesting that increased particle repulsion by citrate and the electrosteric effect of D7 also acted in cell culture medium to partially disperse the NP agglomerates. The combination treatment of citration + D7 was even more effective at reducing agglomerate sizes. However, none of the treatments was able to disperse the HA NP agglomerates down to their primary particle sizes. Mø-SFM does not contain serum, but does contain albumin and growth factor proteins. The possibility that these medium proteins contributed to agglomerate dispersion for all the HA NP species cannot be excluded. Some studies have found that addition of foetal calf serum or human serum can significantly reduce agglomerate sizes of various NPs compared to unsubstituted culture medium, an effect thought to be due to steric stabilization of the NPs by bound serum proteins [31,35]. Taken together, citrate adsorption following the precipitation reaction and the addition of D7 both significantly reduced the average agglomerate sizes of non-autoclaved and autoclaved HA NP in Mø-SFM in our study, a pre-requisite to testing the effect of agglomeration on NP uptake, toxicity and the formation of the SCC.

### 4.2 Effect of agglomerate dispersion on HA NP uptake and cytotoxicity

Exposure of HMMs to 125 μg/ml NANC or ANC led to dramatic increases in calcium uptake as measured by ICP-OES and a significant drop in cell viability in our study. The increased uptake may partly be due to sedimentation of the larger agglomerates onto the cells, resulting in higher local concentrations of NPs than the bulk concentration would imply. To limit the effect of sedimentation (and effects of cytotoxicity), shorter incubation times of 1–2 h were chosen for analysis of Ca-uptake by ICP-OES and SCC formation as opposed to cytotoxicity experiments. Even partial dispersion of HA NP agglomerates by citration or addition of D7 significantly decreased the amounts of cell-associated calcium, with the combination treatment (citration + D7) being the most effective. Concomitantly, all three dispersion treatments prevented HA NP toxicity and returned cell viability back to the level of untreated control cells. This confirms our previous observation, that the observed HMM toxicity is correlated with the degree of HA NP uptake [11]. The observed toxicity is probably due to release of free Ca^2+^ from the HA NPs. This view is supported by the change in particle morphology following uptake observed in some samples by BF-TEM (reduction in size and rounding of the needle- and plate-like shapes of the as-synthesized particles), which we surmise is due to beginning particle degradation. In contrast to the correlation between HA NP uptake and cytotoxicity, there was no simple correlation between agglomerate size and HA NP uptake. Although addition of D7 was more effective than citration in reducing average agglomerate sizes, cell-associated calcium was lower after exposure to citrated NPs than D7-dispersed NPs. Therefore, the quantity of HA NP taken up may not only depend on agglomerate size, but may also be influenced by the surface charge or protein or other coating of the agglomerates.

A vast number of studies have looked at the effect of aggregation/agglomeration on NP uptake, cytotoxicity or other biological effects; indeed too many to give a comprehensive summary. Please refer to Table 2 for an overview of some of these studies. In agreement with us, some studies have found a dependency of NP uptake on agglomeration, sometimes only for certain NP species or for certain cell types [31,34,36,37]. In other studies, the degree of particle uptake appeared to be governed by other parameters – such as the cell type used and the uptake mechanism they engaged, type of particle surface coating and/or particle charge – rather than aggregation [32,38–41]. Yet other studies stated that NP uptake and cytotoxicity were dominated by the specific-surface area, phase composition and crystallinity of the particles [42,43]. From the studies summarized in Table 2, it seems evident that generalizations on the effect of NP aggregation/agglomeration on cellular uptake and biological effects are impossible, but have to be investigated on an individual basis – including the species of NP examined, the composition of the biological medium used for dispersion and the particular cell type studied. Naturally, the biological effect of the NPs – be it cytotoxicity or other – will not only depend on their chemical nature and degree of uptake, but also on the composition of the acquired NP protein corona, intracellular NP localization and biostability in the cellular environment. Our prime interest in this study was to see whether NP agglomeration governed the *mode* of uptake into HMMs, i.e. the internalization of HA NPs into the SCC.

### 4.3 Effect of HA agglomerate dispersion on SCC formation

In previous studies, the formation of the SCC was observed after exposure of macrophages to fairly high concentrations of potentially aggregated materials, such as acetylated LDL, microcrystalline cholesterol, latex, gold, or polystyrene beads. However, particle aggregation was not investigated in these studies and the formation of the SCC was thought to be triggered by the hydrophobicity of the NPs [13–16]. We showed in a previous study that both uncitrated and citrated HA NPs are hydrophilic using a DIW/octanol partition assay, suggesting that SCC formation may not be governed by particle hydrophobicity [12]. However, in view of the fact that many NPs acquire a protein corona when dispersed into cell culture media, the hydrophilicity/hydrophobicity of NPs may need to be evaluated after incubation in cell culture medium. Early indications that aggregation may be the trigger for SCC formation came from studies by Zhang et al., where aggregated LDL could be released from the SCC by treatment with the proteases trypsin or plasmin. Aggregated LDL pre-treated with trypsin was not internalized by the cells, which was thought to be due to the loss of a protein ligand or loss of multi-valency of the aggregated LDL necessary for uptake rather than loss of aggregation state [14]. The aggregated LDL released from the SCC after treatment with plasmin was shown to be partially disaggregated [44]. In a previous study of ours, SCC formation was observed particularly at high NP concentrations, which led to our hypothesis that the formation of the SCC may be triggered by agglomeration. In our study, exposure of HMMs to NANC and ANC particles, which form large agglomerates in Mø-SFM, led to the formation of an extensive SCC already after 2 h and even more so after 24 h. The formation of the SCC was concentration-dependent. It was already visible after exposure of cells to 30 μg/ml NANC or ANC, but its size became much more extensive with increasing NP concentrations. Partial dispersion of agglomerates by citration or the addition of D7 reduced, but not altogether abolished SCC formation. In agreement with an earlier study of ours [12], HA NPs were occasionally located in smaller vesicles, often with a much higher packing density. This could indicate that small NP agglomerates are taken up by mechanisms other than the SCC, such as phagocytosis or endocytosis, smaller vesicles may bud off the SCC, or both. The combination treatment (citration + D7), which was the most effective method at reducing agglomerate sizes, prevented the formation of the SCC altogether and HA NP were only occasionally seen in small vacuoles.

Taken together our data suggest that the formation of the SCC is triggered by the exposure of macrophages to large particle agglomerates. Dispersion of agglomerates prevents the SCC from forming, but does not completely prevent HA NP uptake by other mechanisms.

### 4.4 Role of the SCC *in vivo*?

To our knowledge, the SCC has so far only been observed in cells *in vitro*, and evidence that this compartment exists *in vivo* is still lacking. This is despite the fact that numerous situations exist where cells *in vivo* are exposed to large, particulate aggregates/agglomerates that could favour SCC formation – such as aggregated lipid deposits in atherosclerotic lesions; mineral deposits in ectopic calcification, kidney disease and osteoarthritis; implant wear debris; syndromes characterized by abnormal protein aggregation; to name a few. The reasons that the SCC so far may have been missed could be technical as well as physiological. We used live multiphoton imaging and serial block face SEM and 3D image reconstruction to show that the SCC in macrophages is an extensive, interconnected membrane system that is open to the extracellular space [12]. Traditionally, serial sectioning had to be used to give a three-dimensional impression of cells and tissues, an approach which is both difficult and time-consuming. In addition, the SCC observed in cell culture may be an ‘extreme’ situation, brought about by exposure of cells to extremely high concentrations of particulate agglomerates. As our concentration-dependence experiment has shown, SCC formation at lower particle concentrations is less obvious and could easily be mistaken for other forms of uptake, such as phagocytosis, especially when using traditional 2D techniques. Alternatively, ‘spotting’ the SCC may be made more difficult if it is a transient, short-lived compartment. May be with the advent of faster 3D imaging techniques, the discovery of the SCC in cells *in vivo*, especially in the above mentioned disease situations, is awaiting.

Assuming the SCC existed *in vivo*, what function could it serve? The work by Kruth and others suggested that the SCC enabled macrophages to sequester large amounts of hydrophobic particulate material from the surrounding tissue and then slowly degrade it [13–16]. This was thought to be important in the atherosclerotic lesion, where aggregated LDL – generated by oxidative processes, enzymatic action or binding to exposed extracellular matrix molecules – is deposited in the intima of the vessel wall. Although the initial internalization of aggregated LDL into the SCC was independent of the LDL-receptor, slow LDL receptor-dependent degradation of the internalized LDL was taking place [14]. In addition, the macrophages were able to disaggregate the sequestered aggregated LDL through the action of plasmin activation. Upon release from the SCC, the released material was only partially aggregated and contained larger lipid particles, a sign that LDL fusion had occurred. This was thought to be the mechanism that transformed LDL into the structures size-consistent with lipid particles found in atherosclerotic lesions [44]. We propose SCC formation may be a protective mechanism, an attempt to ‘shield’ surrounding cells and tissues from damage by rapid sequestration of particle agglomerates/aggregates. Our work also suggests that the SCC is not inert and that particle degradation is taking place, although the mechanism by which this happens is unclear. The macrophages in our *in vitro* experiments eventually die, probably brought about by the free calcium released from the HA NPs [11,12]. However, *in vivo* the released calcium may be free to diffuse into the vast volume of the interstitial fluid without inducing vast toxicity to the macrophages themselves and to the surrounding cells.

So if the formation of the SCC is a protective mechanism, what is it trying to protect from? It has been known for decades that the macrophage is part of the innate immune system, continuously patrolling tissues to differentiate between ‘self’ and ‘non-self’ and to deal with any offenders, be it foreign particles, tumour cells, denatured proteins or hostile pathogens. May be the formation of the SCC is a mechanism to rapidly remove large amounts of any ‘suspicious’ agglomerated/aggregated material from the tissue, before ‘deciding’ what action to take: be it proteolytic digestion, dissolution by acidification, killing using reactive oxygen intermediates or raising an appropriate immune response [45]. There is a vast amount of literature indicating that particulate agglomerates *in vivo* can be highly inflammatory [4,46,47]. The NLRP3 inflammasome has now been shown to be activated by endogenous particulates, such as uric acid and cholesterol crystals, as well as by anthropogenic materials, such as asbestos and silica [22,45,48,49]. Synthetic HA particles of a fine needle shape or HA aggregates, resembling HA crystals found in synovium, can activate the NLRP3 inflammasome in LPS-primed macrophages and lead to increased secretion of the pro-inflammatory cytokines IL-1β and IL-18. In turn, these pro-inflammatory cytokines are thought to mediate the synovial inflammation and cartilage degeneration seen in osteoarthritis [50]. Micro-calcification (‘spotty’ calcification) associated with progressive inflammation is more prone to result in the destabilization of atherosclerotic lesions and myocardial infarction than advanced macro-calcification [6].

It will be highly informative to investigate the functional dynamics of the SCC, the origin and protein/receptor profile of its membrane, and its inflammatory ‘status’. Interestingly, a surface-connected tubular compartment has recently been identified in HIV-infected macrophages [51,52]. This open-ended compartment is connected to the extracellular space by narrow membrane channels and is now thought to act as a reservoir, enabling the macrophage to release infectious HIV virions to persistently re-infect bystander T-cells. The authors suggested that this compartment may be related to other tubulovesicular compartments observed in antigen-presenting cells – normally functioning in antigen capture, processing, sequestration and presentation – and is ‘hijacked’ by HIV to hide virions intracellularly from the immune response [51,52]. It is unknown at present, how the SCC compares functionally to these other tubulovesicular compartments.

## 5 Conclusions

Our study has shown that HA NPs are internalized extensively by human macrophages resulting in dose-dependent cytotoxicity. At high concentrations, the HA NPs were found sequestered within a highly branched, interconnected compartment that is open to the extracellular space. The formation of this surface-connected compartment (SCC) was governed by NP agglomeration. Dispersion of the HA NP agglomerates by surface citration, addition of the dispersant D7, or a combination of both treatments inhibited the formation of the SCC, significantly reduced the cell-associated calcium load and prevented HA NP cytotoxicity. In BF-TEM imaging HA NPs showed signs of degradation within the SCC, suggesting that it is not an ‘inert’ compartment. The inhibition of SCC formation did not preclude HA NP uptake on a small scale by other mechanisms. We propose that the formation of the SCC is a protective mechanism, ‘shielding’ surrounding cells and tissues from damage by rapid sequestration of large particle agglomerates. Future studies should concentrate on exploring the functional dynamics of the SCC and its role in inflammatory responses to NP agglomerates. This would be highly relevant to understanding the interactions of HA NPs with cells in normal developmental/pathological processes and in biomedical applications of HA vectors.

## Figures and Tables

**Table 1:** Primary particle characteristics of HA NPs.

HA NP	Particle dimensions^a^	Aspect ratio	Zeta potential (−mV)^b^		Surface area (m^2^/g)^c^
			−D7	+D7	
NANC	L: 30 ± 5 nm S: 8 ± 2 nm	3.9 ± 1.1	−17.80 ± 1.42	−36.11 ± 2.98	121.4 ± 0.6
NAC	L: 27 ± 7 nm S: 8 ± 2 nm	3.6 ± 1.2	−14.48 ± 0.92	−24.08 ± 1.29	156.4 ± 1.5
ANC	L: 54 ± 19 nm S: 18 ± 5 nm	3.2 ± 1.2	−10.56 ± 0.84	−55.84 ± 1.20	64.9 ± 0.35
AC	L: 46 ± 18 nm S: 17 ± 5 nm	2.8 ± 0.95	−24.29 ± 0.75	−43.60 ± 2.48	52.8 ± 0.3

Particle dimensions and aspect ratios are means ± SD (*n* = 100); L = long axis; S = short axis.

a Determined by BF-TEM.

b Determined by electrophoretic mobility measurement in 5 mm KNO_3_ at pH 7.0 and 20 °C (mean ± SE).

c Determined by BET-measurement.

**Table 2:** Studies on the effect of NP agglomeration/aggregation on cellular particle uptake and cytotoxicity.

NP species	Cell type	Results	Ref.
Al, Al_2_O_3_, Ag, Cu, TiO_2_, SiO_2_ of different sizes	HEL-30 mouse keratinocytes	Aggregation of NPs increased cytotoxicity for some NP types, but not for others.	[31]
TiO_2_ of different sizes	THP-1 human monocytic cells, NCI-H292 human bronchial epithelial cells	No difference in cytotoxicity between small and large NP aggregates in THP-1 cells. In NCI-H292 cells large particle aggregates are more toxic.	[36]
Fe_2_O_3_ and CeO_2_ with various functionalizations	NIH/3T3 mouse fibroblasts	Degree of NP uptake is a function of aggregate size and type of surface functionalization.	[37]
PVA-coated SPION (superparamagnetic iron oxide NPs)	HeLa human cervix carcinoma cells	Increasing aggregation favours enhanced uptake. This effect was not driven by sedimentation as aggregates were stable in suspension.	[34]
Transferrin-coated Au NPs	HeLa, A549 human lung epithelial carcinoma cells, MDA-MB-435	Aggregation leads to decreased uptake in HeLa and A549 cells, but to an increase in MDA-MB-435 cells. Uptake is dependent on cell type and uptake mechanism used.	[38]
Ag and TiO_2_ NPs	HepG-2 human hepatoma cells, THP-1, A549	The least agglomerated particles are the most cytotoxic.	[39]
Ag NPs with various functionalizations	RAW-264.7 mouse macrophages, C-10 lung epithelial cells	NP cytotoxicity depends on the type of surface coating, particle charge, extent of aggregation and cell type used.	[40]
SiO_2_ NPs with various functionalizations	HaCaT human keratinocytes, primary skin cells	Degree of NP uptake is independent of aggregate size, but dependent on particle charge in HaCaT cells. Aggregation leads to a decrease in particle uptake in primary skin cells.	[41]
SiO_2_ NPs with various functionalizations	HeLa human cervix carcinoma cells	Efficient cellular NP uptake is favoured by high colloidal stability in cell culture medium and a high positive zeta potential. Aggregates bind to the cells, but are not internalized.	[32]
Monodisperse and aggregated silicone-based NPs	J774 mouse macrophages, BALB/c3T3 mouse fibroblasts	Cytotoxicity of NPs is dependent on particle surface area rather than aggregation status.	[42]
TiO_2_ anatase and anatase/rutile NPs	Caco-2 human colon adenocarcinoma cells	Cytotoxicity is determined by the specific surface area, crystallinity and phase composition of the particles.	[43]

## Appendix A Supplementary data

Table SI1: Average agglomerate sizes of HA NP preparations in DIW and Mø-SFM as measured by DLS

Fig. SI1: Phase identification of NANC and ANC by powder XRD. The peaks in the NANC and ANC spectra are matched by the peak list of a synthetic hydroxyapatite from the reference data base (ASTM card 00-009-0432).

Fig. SI2: Powder XRD. Overlay of spectra from A) NANC and ANC and B) NANC and NAC, showing the effects of A) autoclaving and B) citration on peak shape and position.

Fig. SI3: Adsorption and desorption isotherms of NANC and ANC (N_2_ analysis adsorptive, at 77 K in liquid nitrogen). The table lists the mean BJH surface area of pores and mean pore diameters for all four HA powders.

Fig. SI4: SEM of HA NP agglomerates. HA NPs were suspended in Mø-SFM at 125 μg/ml in the presence of 0.125% D7 and incubated overnight. Then, suspensions were passed through membrane filters with 3 μm pores. Subsequently, filters were prepared for SEM. A) NANC; B) ANC; C) NAC; D) AC; E) NANC –detail; F) ANC – detail.

Fig. SI5: Concentration-dependence of SCC formation. HMMs were incubated for 24 h with A) 30 μg/ml, B) 60 μg/ml or C) 125 μg/ml NANC (left column) or with D) 30 μg/ml, E) 60 μg/ml or F) 125 μg/ml ANC (right column); scale bars are 2 μm

Fig. SI6: BF-TEM – A) untreated control cells; B) cells treated with 0.125% D7 only for 24 h. Scale bars are 2 μm.

Fig. SI7: BF-TEM of HA NP degradation within the SCC – Overviews. HMMs were incubated with 125 μg/ml NANC (A) or ANC (B) for 2 h prior to TEM processing. Scale bars are 2 μm. HA NPs are contained within the SCC and the areas within the squares are shown in greater detail in Fig. 7 of the main manuscript.
