# Supplementary material for: The effect of particle agglomeration on the formation of a surface-connected compartment induced by hydroxyapatite nanoparticles in human monocyte-derived macrophages
Source: Biomaterials. 2014 Jan;35(3):1074–88. doi: 10.1016/j.biomaterials.2013.10.041 (PMC3843813; doi:10.1016/j.biomaterials.2013.10.041)
Supplement: Supplementary file 1 — Table SI1: Average agglomerate sizes of HA NP preparations in DIW and Mø-SFM as measured by DLS [file mmc1.docx]

Table SI1: Average agglomerate sizes of HA NP preparations by DLS

| (in nm) | | DLS |
| --- | --- | --- |
|  | **D7** | **DIW** |
| NANC | **-** | **ND** |
|  | **+** | **172 ± 2** |
| NAC | **-** | **131 ± 17.5** |
|  | **+** | **145 ± 5** |
| ANC | **-** | **2347 ± 96** |
|  | **+** | **249 ± 2** |
| AC | **-** | **160 ± 6** |
|  | **+** | **218 ± 2** |

*Values are means ± SD (n=6); measured at
pH 7.0 and 20^o^C. ND = not determined as
NANC in DIW agglomerated too quickly as to
interfere with the measurement.*
