# Supplementary figures and images for: The effect of particle agglomeration on the formation of a surface-connected compartment induced by hydroxyapatite nanoparticles in human monocyte-derived macrophages
Source: Biomaterials. 2014 Jan;35(3):1074–88. doi: 10.1016/j.biomaterials.2013.10.041 (PMC3843813; doi:10.1016/j.biomaterials.2013.10.041)

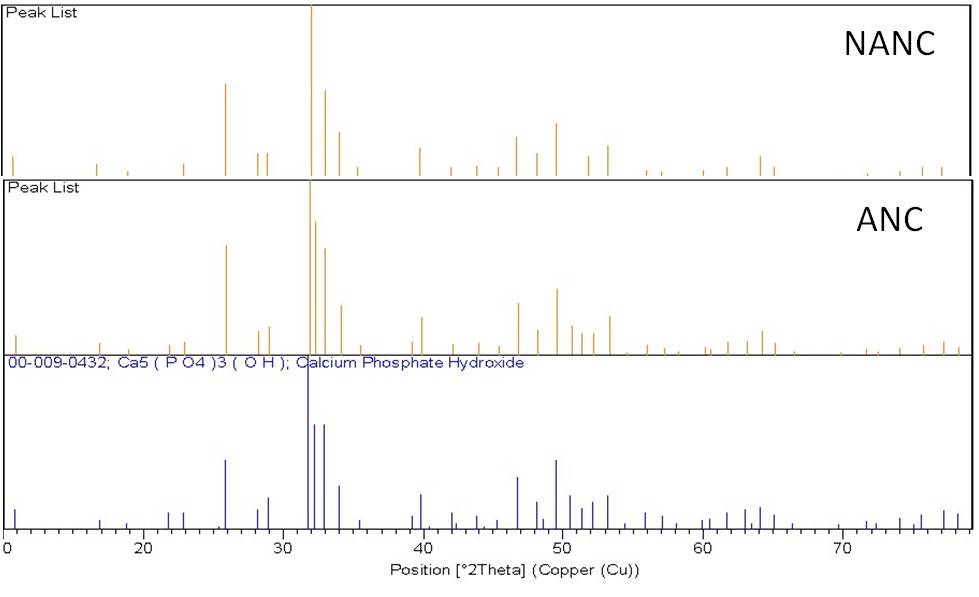

Supplement: Supplementary file 2 — Fig. SI1: Phase identification of NANC and ANC by powder XRD. The peaks in the NANC and ANC spectra are matched by the peak list of a synthetic hydroxyapatite from the reference data base (ASTM card 00-009-0432). [file mmc2.docx]

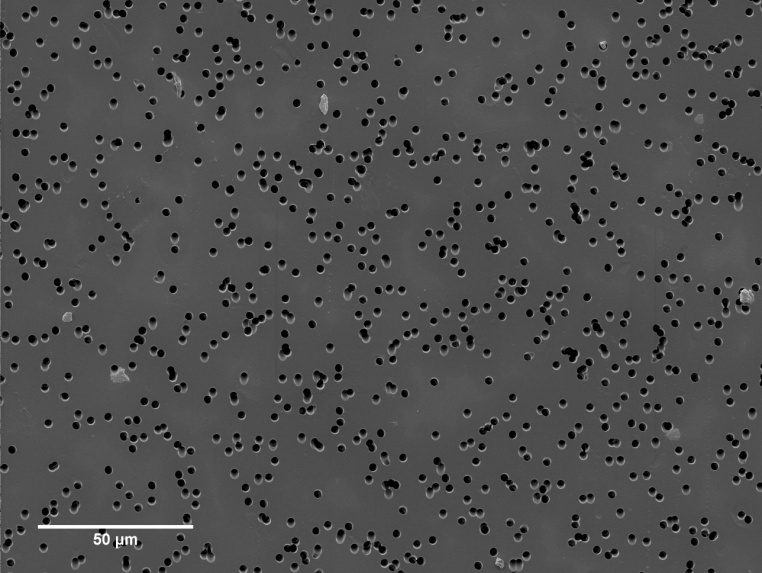

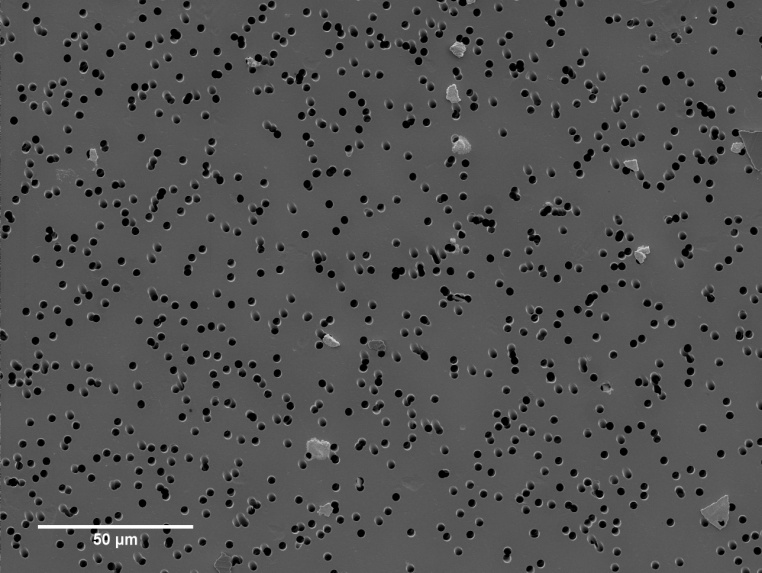


**B**

**A**


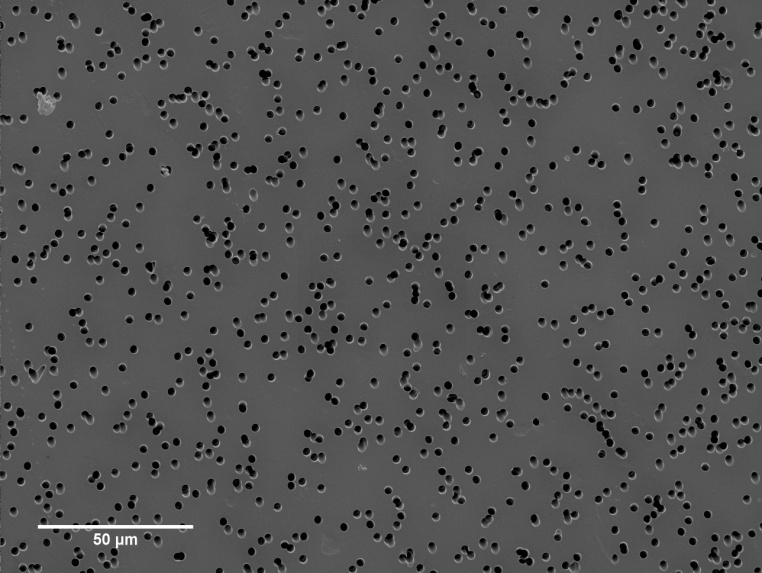

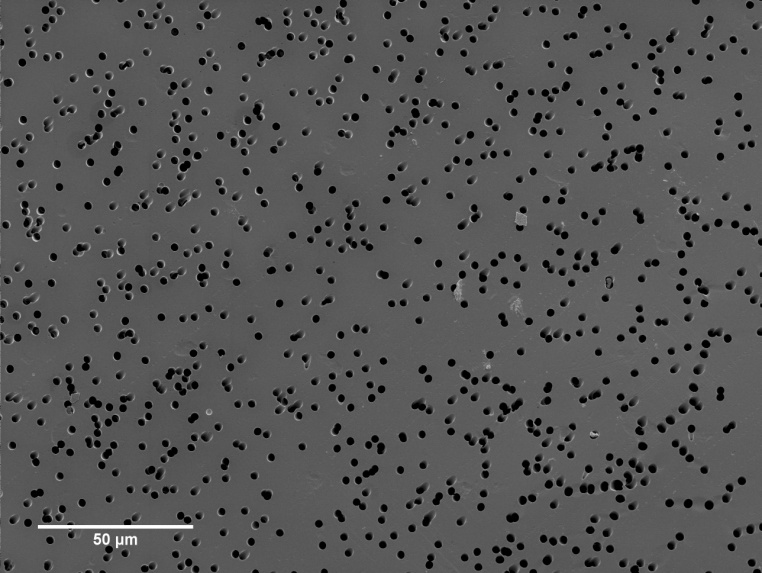


**D**

**C**


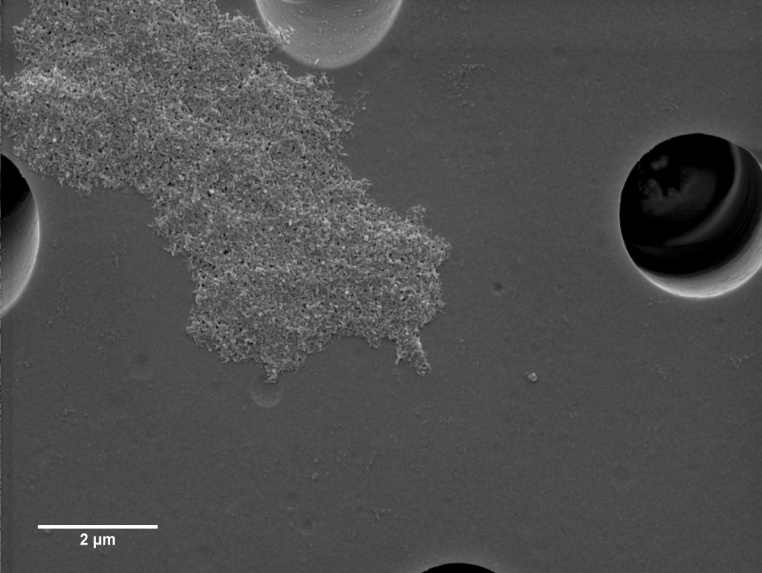

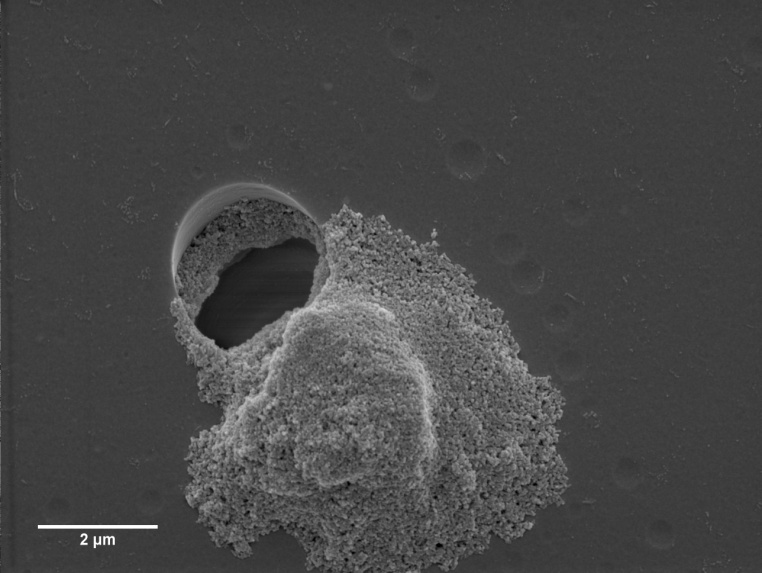


**F**

**E**

Supplement: Supplementary file 5 — Fig. SI4: SEM of HA NP agglomerates. HA NPs were suspended in Mø-SFM at 125 μg/ml in the presence of 0.125% D7 and incubated overnight. Then, suspensions were passed through membrane filters with 3 μm pores. Subsequently, filters were prepared for SEM. A) NANC; B) ANC; C) NAC; D) AC; E) NANC –detail; F) ANC – detail. [file mmc5.docx]

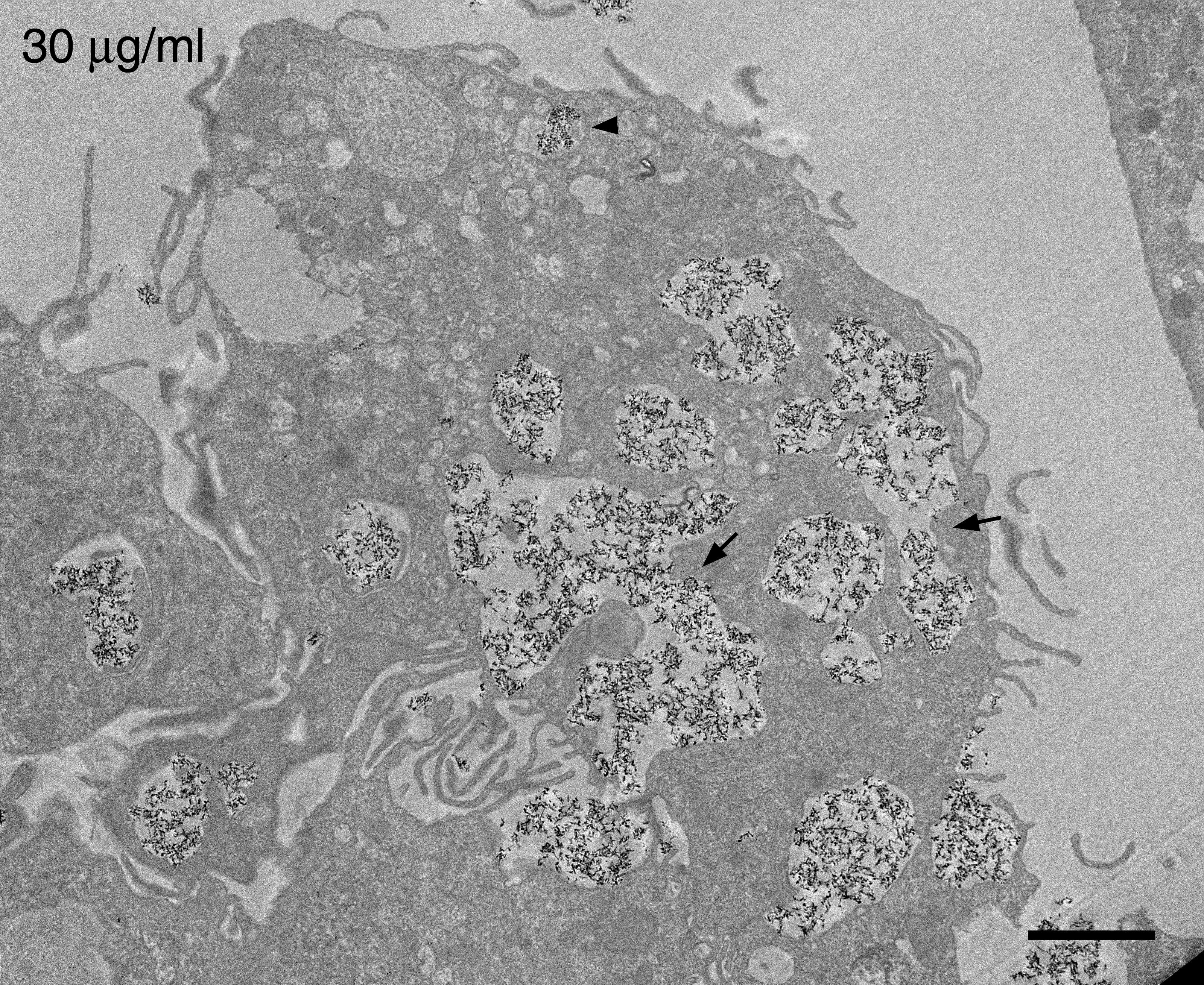

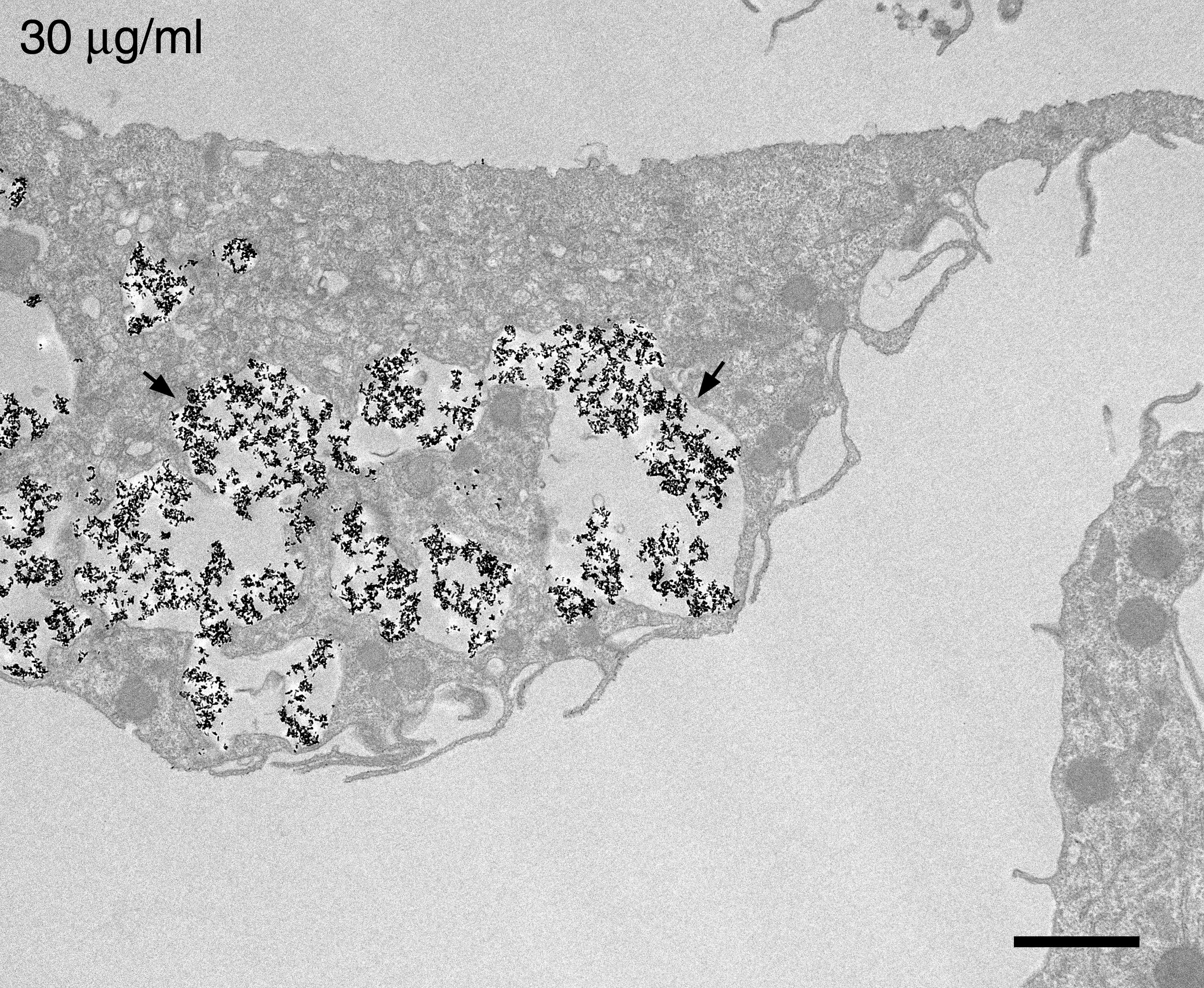


**NANC**

**ANC**

**D**

**D**

**A**


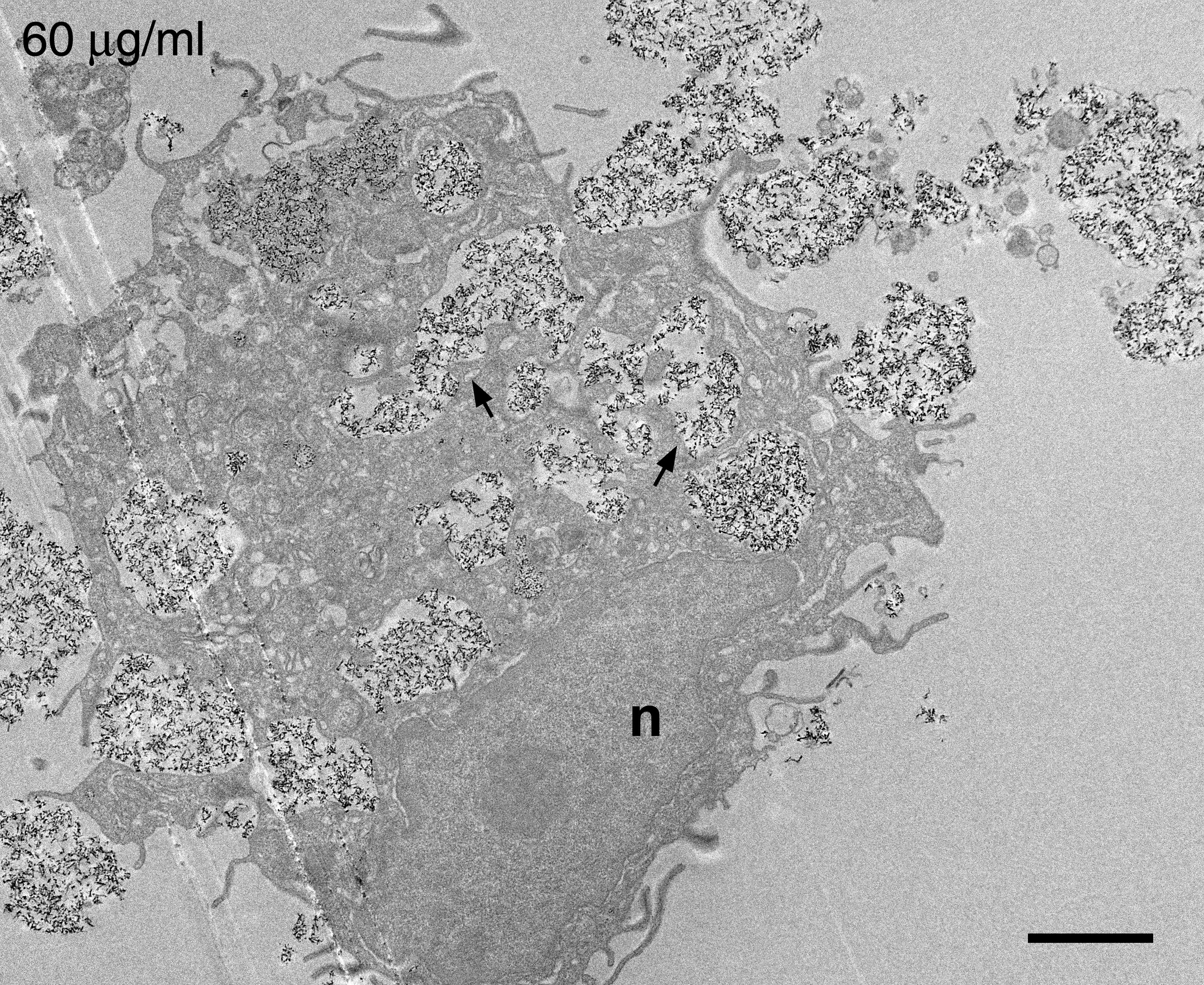

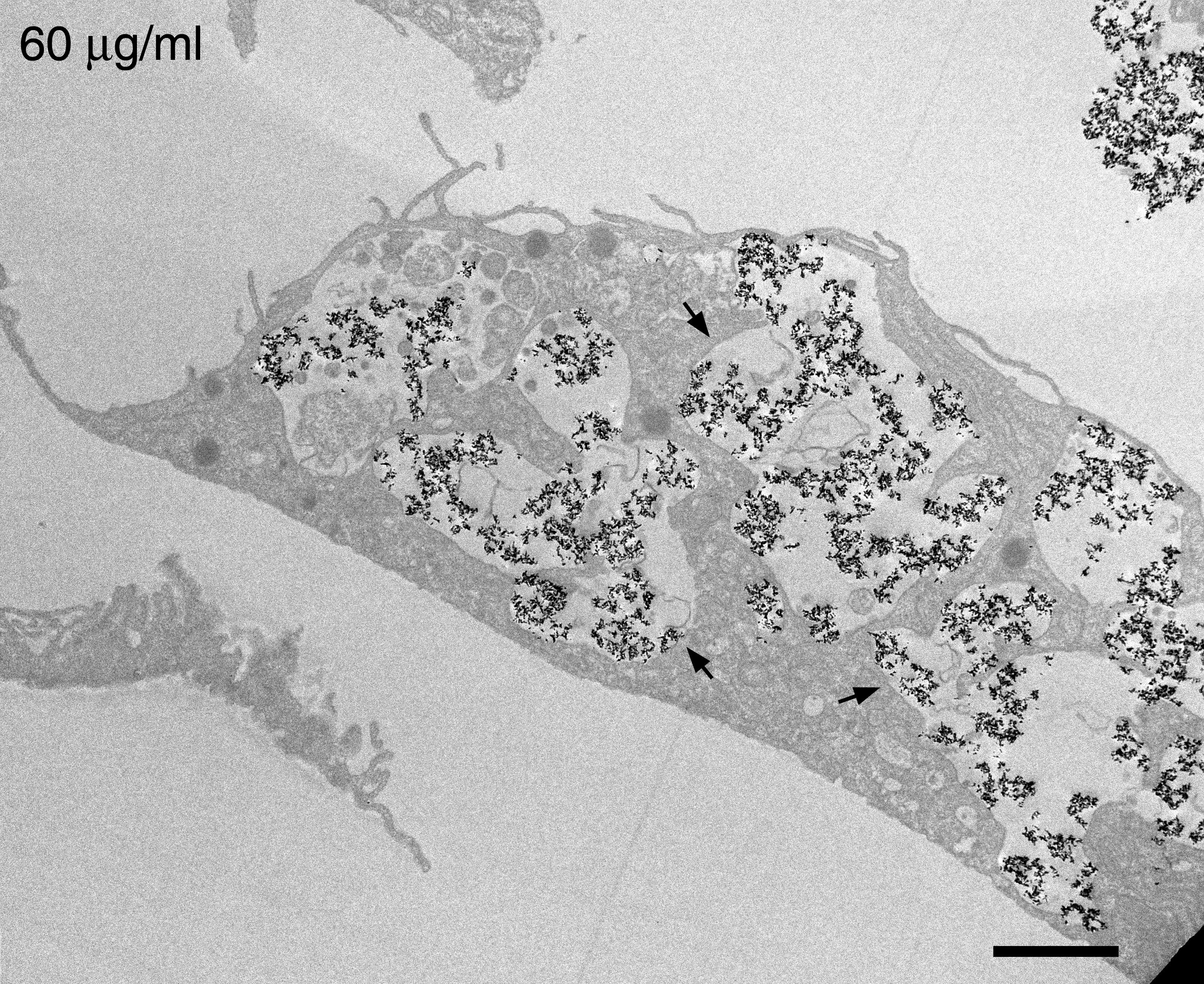


**E**

**B**


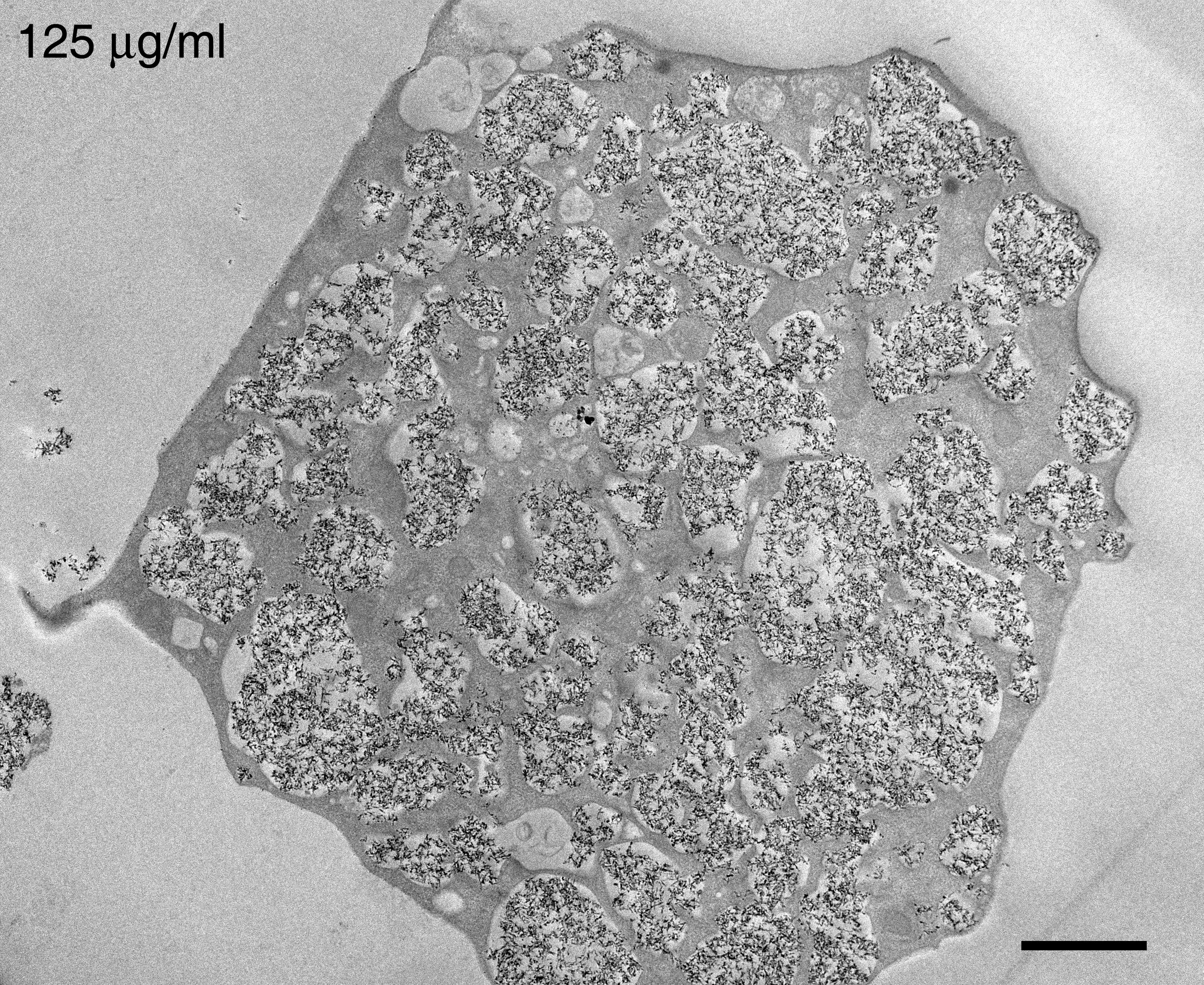

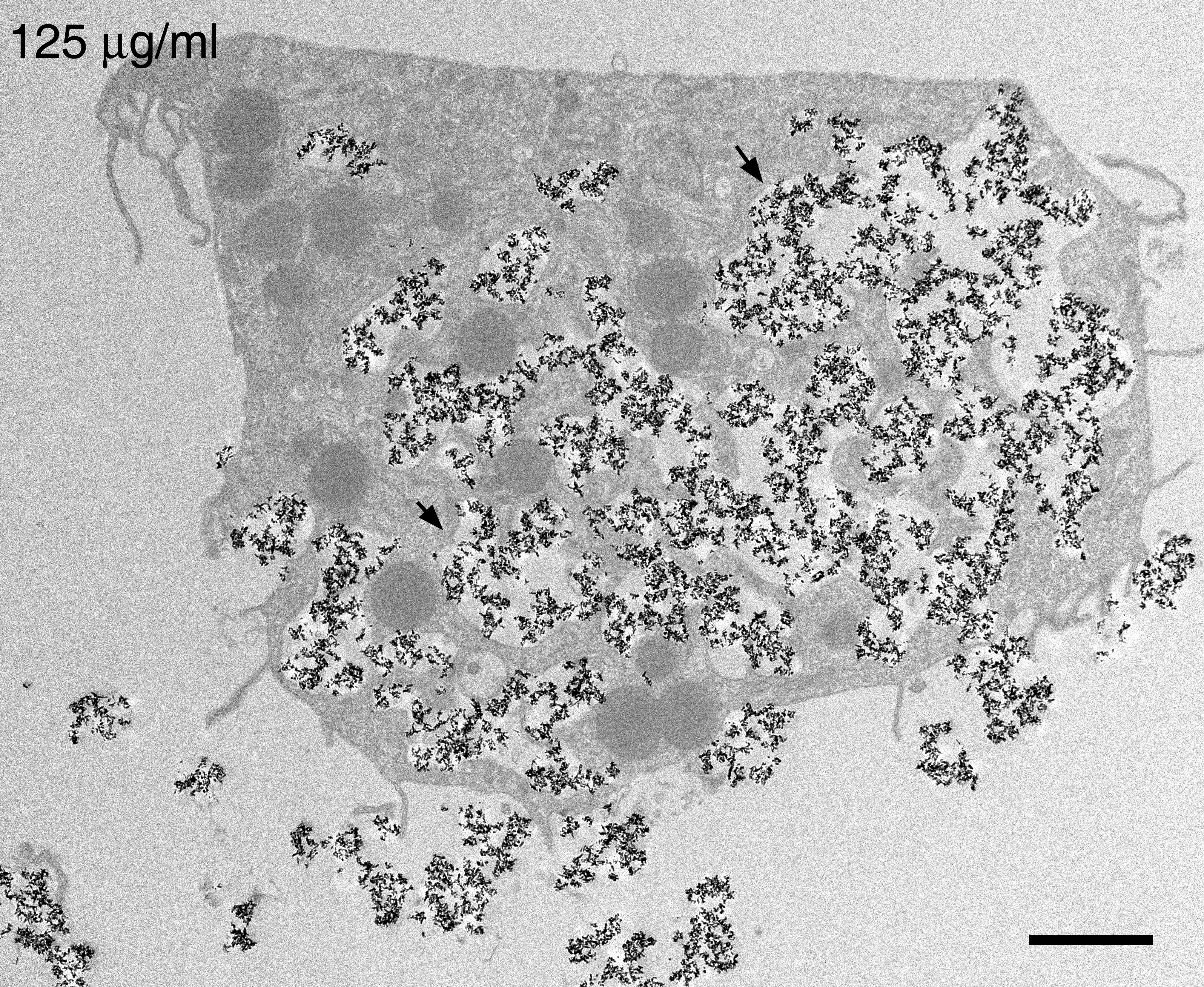


**F**

**C**

**D**

Supplement: Supplementary file 6 — Fig. SI5: Concentration-dependence of SCC formation. HMMs were incubated for 24 h with A) 30 μg/ml, B) 60 μg/ml or C) 125 μg/ml NANC (left column) or with D) 30 μg/ml, E) 60 μg/ml or F) 125 μg/ml ANC (right column); scale bars are 2 μm [file mmc6.docx]

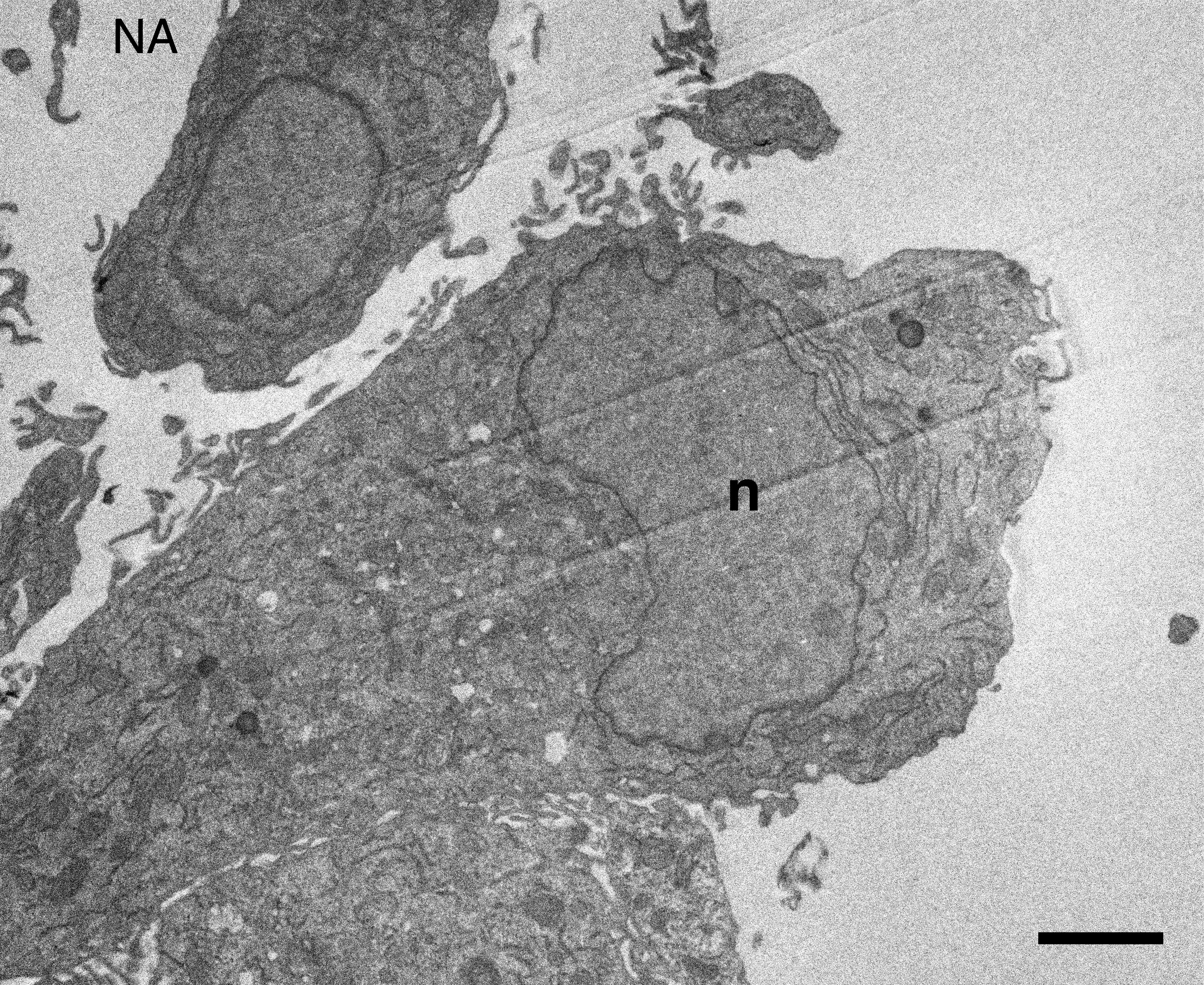

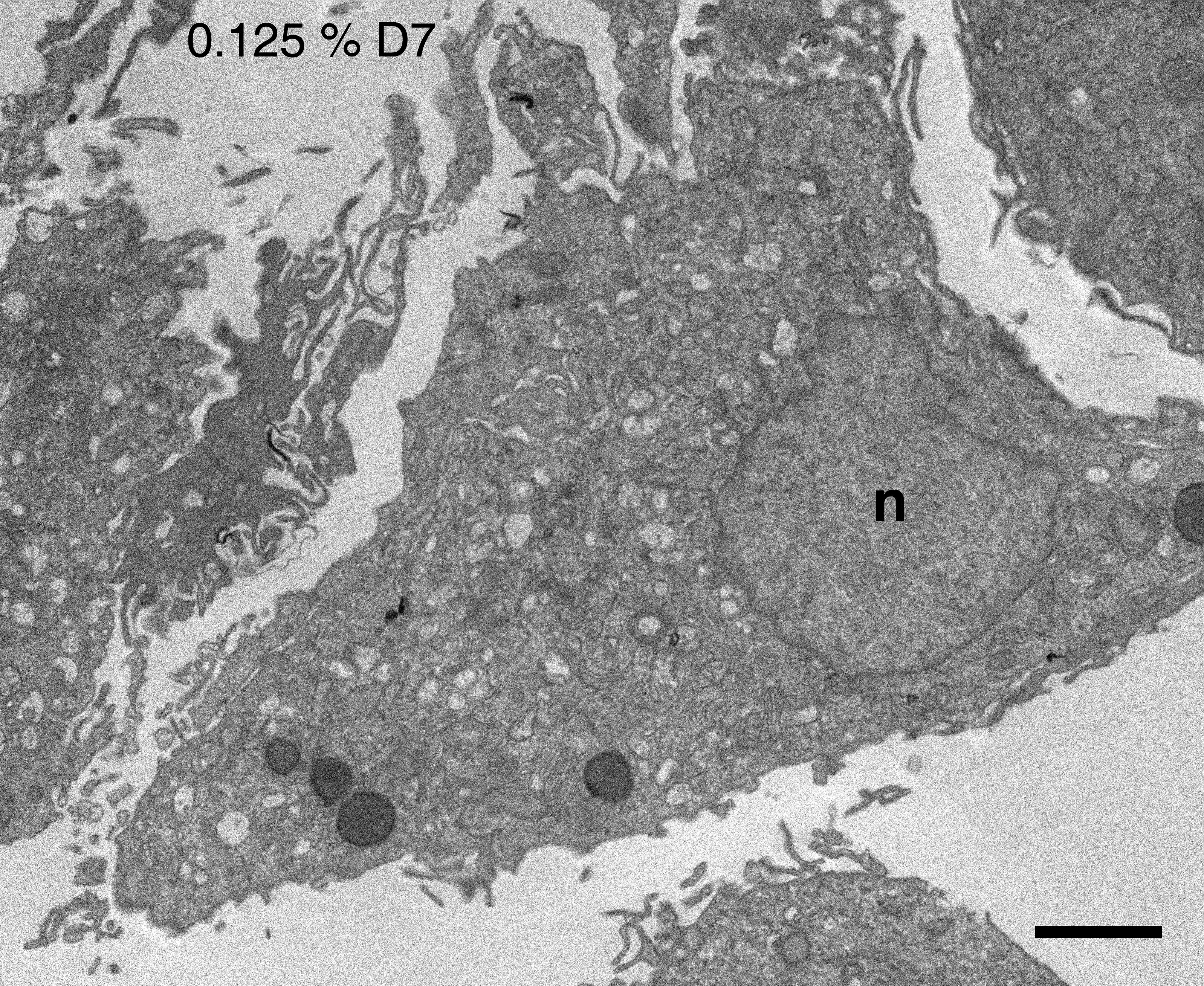


**B**

**A**

Supplement: Supplementary file 7 — Fig. SI6: BF-TEM – A) untreated control cells; B) cells treated with 0.125% D7 only for 24 h. Scale bars are 2 μm. [file mmc7.docx]

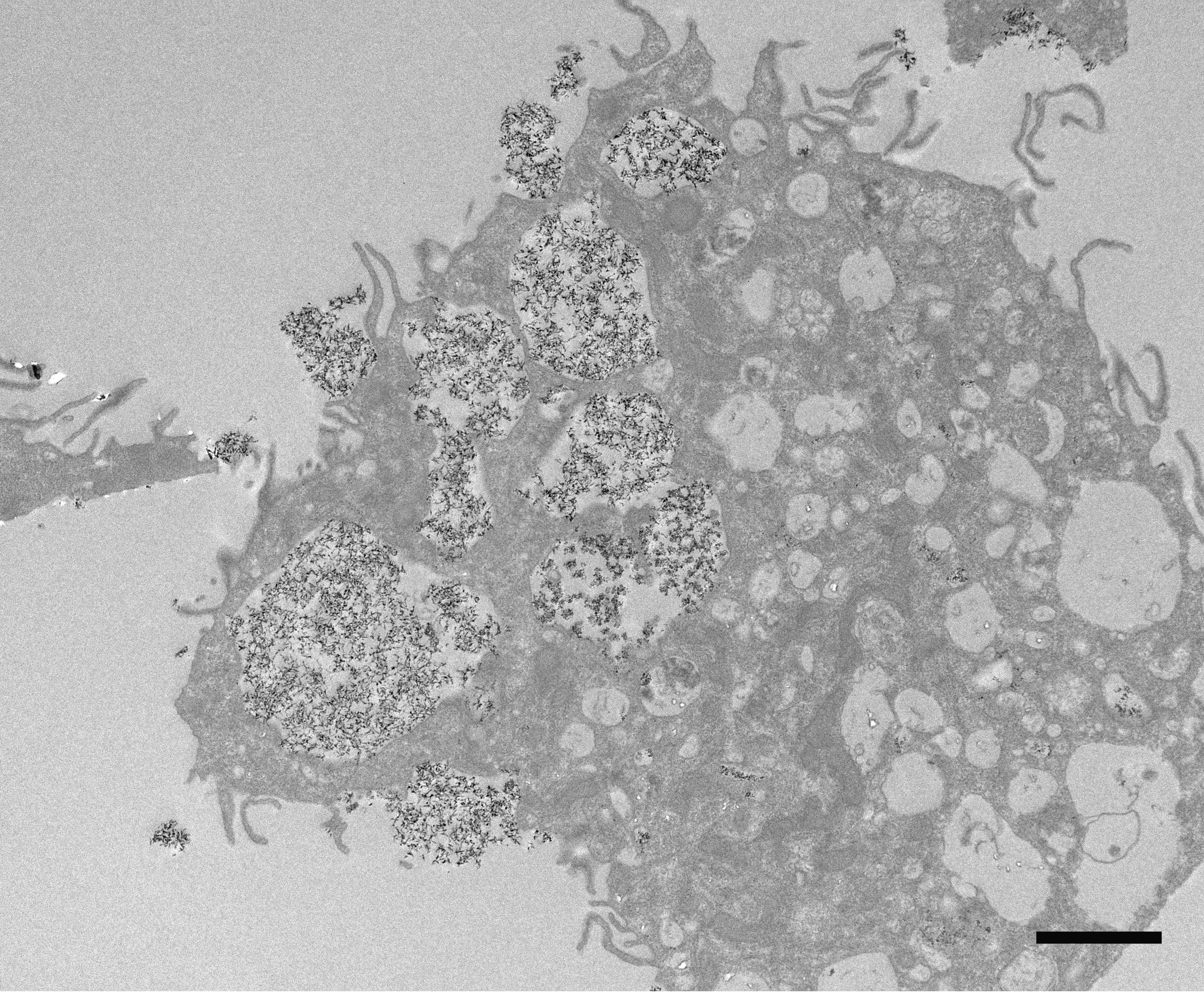

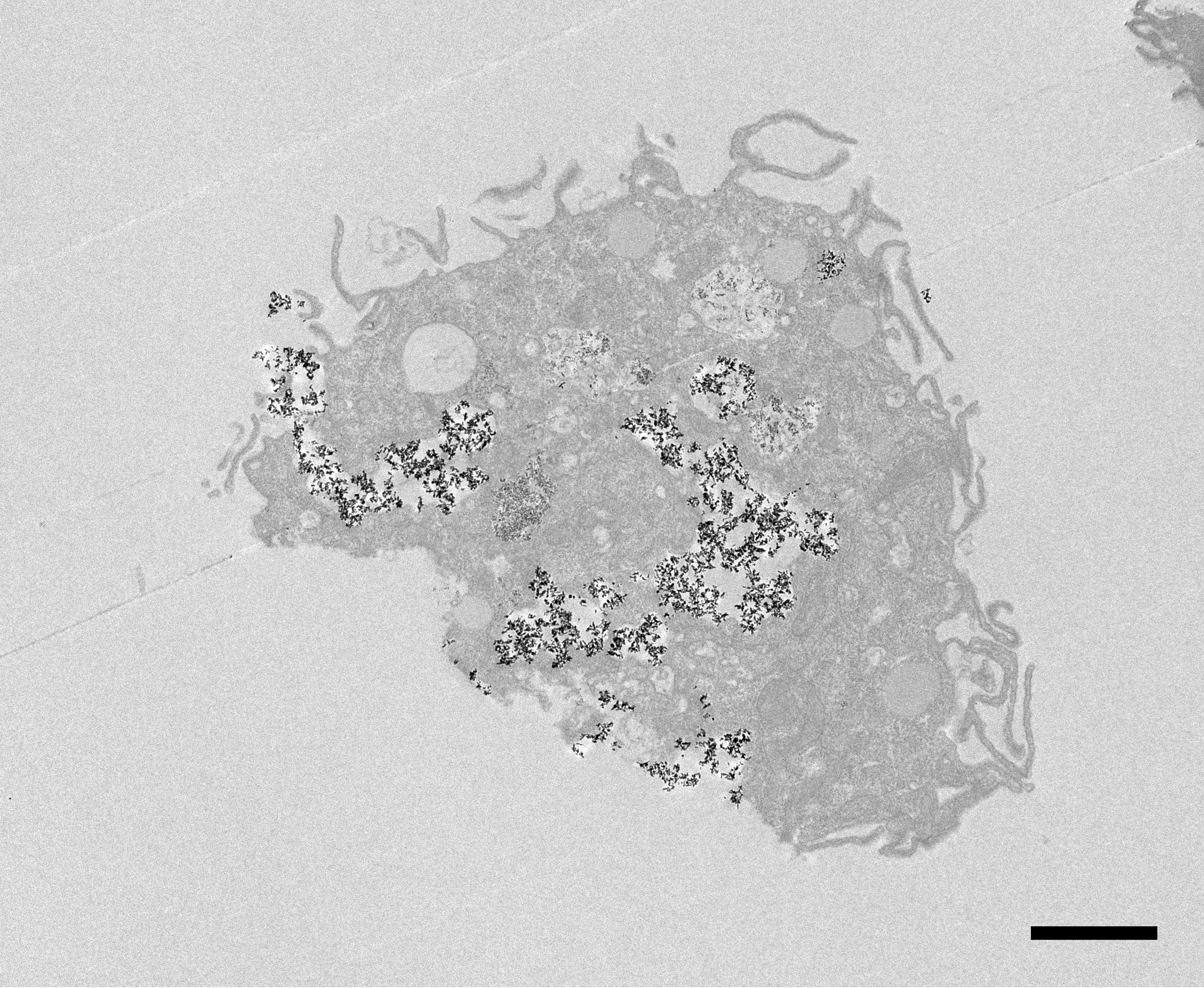


**B**

**A**

Supplement: Supplementary file 8 — Fig. SI7: BF-TEM of HA NP degradation within the SCC – Overviews. HMMs were incubated with 125 μg/ml NANC (A) or ANC (B) for 2 h prior to TEM processing. Scale bars are 2 μm. HA NPs are contained within the SCC and the areas within the squares are shown in greater detail in Fig. 7 of the main manuscript. [file mmc8.docx]
