# Supplementary material for: The effect of particle agglomeration on the formation of a surface-connected compartment induced by hydroxyapatite nanoparticles in human monocyte-derived macrophages
Source: Biomaterials. 2014 Jan;35(3):1074–88. doi: 10.1016/j.biomaterials.2013.10.041 (PMC3843813; doi:10.1016/j.biomaterials.2013.10.041)
Supplement: Supplementary file 4 — Fig. SI3: Adsorption and desorption isotherms of NANC and ANC (N2 analysis adsorptive, at 77 K in liquid nitrogen). The table lists the mean BJH surface area of pores and mean pore diameters for all four HA powders. [file mmc4.docx]

|  | NANC | NAC | ANC | AC |
| --- | --- | --- | --- | --- |
| mean BJH surface area of pores (m^2^/g) | **151.5 ± 11.4** | **204.4 ± 13.8** | **79.45 ± 4.8** | **70.9 ± 9.2** |
| mean BJH pore diameter (nm) | **11.9 ± 0.01** | **7.5 ± 0.5** | **16.2 ± 1.0** | **12.5 ± 1.6** |
| *results given are means ± SD of BJH adsorption and desorption values* | | | | |
