# Supplementary material for: The effect of particle agglomeration on the formation of a surface-connected compartment induced by hydroxyapatite nanoparticles in human monocyte-derived macrophages
Source: Biomaterials. 2014 Jan;35(3):1074–88. doi: 10.1016/j.biomaterials.2013.10.041 (PMC3843813; doi:10.1016/j.biomaterials.2013.10.041)
Supplement: Supplementary file 9 [file mmc9.docx]

**Supplementary Information**

**Results**

*Powder XRD*

All four HA NP powders were analysed by powder XRD between the angles of 2θ of 10^o^ and 80^o^. Figure SI1 shows the result for the phase identification of NANC and ANC with a synthetic HA (A.S.T.M. card 00-009-0432) from the reference database. All peaks in the NANC and ANC spectra were matched by this compound leaving no un-identified peaks. This suggests that NANC and ANC were hydroxyapatite and did not contain any other calcium phosphate phases.

The effect of autoclaving on the HA NPs is shown in Figure SI2A. The XRD spectrum of the autoclaved HA (ANC) shows a much sharper peak pattern than non-autoclaved HA (NANC). This indicates that autoclaving at 120^o^C had increased the crystallite size of ANC and may have also reduced the degree of strain. Figure SI2B overlays the XRD spectra of NANC and NAC. The close match of the two spectra shows that citration had no dramatic effect on crystallite sizes.

*BET/BJH measurements*

All four HA NP powders were analysed using the BET/BJH methods. The shape of the hysteresis in the adsorption/desorption isotherms is characteristic for mesoporous materials (see Figure SI3 for NANC and ANC). The mean pore diameters are in the range of 7.5 nm to 16.2 nm, which falls within the mesoporous range. The non-autoclaved HA NP powders had about 2 -3 times the pore surface area than the autoclaved HA NP powders (Figure SI3, table).

*HA NP agglomerate sizes*

Table SI1 summarizes the average agglomerate sizes of HA NPs in DIW and Mø-SFM in the absence or presence of 0.125 % D7. When compared to cell culture medium, both NANC and ANC form even larger agglomerates in DIW. Citration works better to disperse the agglomerates in DIW than in medium, whereas addition of D7 disperses equally well in DIW and Mø-SFM.

In addition to DLS, we used a SEM/filtration method to examine HA NP agglomerate sizes in cell culture medium. HA NPs were dispersed in Mø-SFM at a concentration of 125 μg/ml in the presence of 0.125 % D7, incubated overnight, mixed briefly and then passed through a membrane filter with 3 mm pores (Figure SI4). In the presence of D7 all four HA NP species hardly left any agglomerates on the filter (Figure Si4A-D). When, isolated agglomerates were located, the primary particles within the agglomerates were clearly visible (Figure SI4D, F).

*Formation of the SCC at different HA NP concentrations – 24h exposure*

Exposure to 30, 60 or 125 µg/ml NANC or ANC for 24h led to increasing particle sequestration by the HMMs (Figure SI5). NPs were contained within a membrane-bounded compartment (arrows), which often appeared inter-connected (SCC). With increasing concentrations, the SCC occupied ever larger proportions of the cells. Occasionally, smaller, isolated vacuoles with more densely packed NPs were visible (Figure SI5A, arrowhead).

*Effect of D7 on HA NP internalization by HMMs – 24h time-course*

The effect of D7 and citration on the uptake of HA NPs in HMMs was studied by BF-TEM after 24h incubation. Untreated control cells and cells treated with 0.125 % D7 only showed normal cellular morphology and no signs of cell damage (Figure SI6 A and B, respectively).

*Degradation of HA NPs within the SCC*

Figure SI7 shows low magnification overview images of HMMs incubated with NANC (A) and ANC (B) for 2h. Both types of HA NP are contained within interconnected, branched membrane-bounded compartments, most likely SCC. The degradation of HA NP is observed in the areas delineated by the black squares. These areas are shown at higher magnifications in the main text of the manuscript.
